# Insulin receptor substrate 1, but not IRS2, plays a dominant role in regulating pancreatic alpha cell function in mice

**DOI:** 10.1016/j.jbc.2021.100646

**Published:** 2021-04-09

**Authors:** Tomozumi Takatani, Jun Shirakawa, Kimitaka Shibue, Manoj K. Gupta, Hyunki Kim, Shusheng Lu, Jiang Hu, Morris F. White, Robert T. Kennedy, Rohit N. Kulkarni

**Affiliations:** 1 Islet Cell and Regenerative Biology, Joslin Diabetes Center, Department of Medicine, Brigham and Women’s Hospital, Harvard Medical School, Boston, Massachusetts, USA; 2 Department of Pediatrics, Graduate School of Medicine, Chiba University, Chiba, Japan; 3 Laboratory of Diabetes and Metabolic Disorders, Institute for Molecular and Cellular Regulation (IMCR), Gunma University, Gunma, Japan; 4 Cell Therapy Translational Engine (CTTE), Takeda Pharmaceuticals, Cambridge, Massachusetts, USA; 5 Departments of Chemistry and Pharmacology, University of Michigan, Ann Arbor, Michigan, USA; 6 Department of Pediatrics, Boston Children’s Hospital, Harvard Medical School, Boston, Massachusetts, USA; 7 Harvard Stem Cell Institute, Harvard Medical School, Boston, Massachusetts, USA

**Keywords:** IRS1, pancreatic alpha cell, pancreatic islet, insulin receptor substrate 1, glucagon, type 2 diabetes, insulin receptor, metabolic disorder, glycemic control, peptide secretion, alphaIRS1KD, IRS1 knockdown, alphaIRS2KD, IRS2 knockdown, alphaIRS1KO, alpha cell-specific IRS1-knockout, BrdU, bromodeoxyuridine, CHX, cycloheximide, IRS1, insulin receptor substrate 1, IRS2, insulin receptor substrate 2, lphaIRS2KO, alpha cell-specific IRS2 knockout

## Abstract

Dysregulated glucagon secretion deteriorates glycemic control in type 1 and type 2 diabetes. Although insulin is known to regulate glucagon secretion *via* its cognate receptor (insulin receptor, INSR) in pancreatic alpha cells, the role of downstream proteins and signaling pathways underlying insulin’s activities are not fully defined. Using *in vivo* (knockout) and *in vitro* (knockdown) studies targeting insulin receptor substrate (IRS) proteins, we compared the relative roles of IRS1 and IRS2 in regulating alpha cell function. Alpha cell–specific IRS1-knockout mice exhibited glucose intolerance and inappropriate glucagon suppression during glucose tolerance tests. In contrast, alpha cell–specific IRS2-knockout animals manifested normal glucose tolerance and suppression of glucagon secretion after glucose administration. Alpha cell lines with stable IRS1 knockdown could not repress glucagon mRNA expression and exhibited a reduction in phosphorylation of AKT Ser/Thr kinase (AKT, at Ser-473 and Thr-308). AlphaIRS1KD cells also displayed suppressed global protein translation, including reduced glucagon expression, impaired cytoplasmic Ca2+ response, and mitochondrial dysfunction. This was supported by the identification of novel IRS1-specific downstream target genes, *Trpc3* and *Cartpt*, that are associated with glucagon regulation in alpha cells. These results provide evidence that IRS1, rather than IRS2, is a dominant regulator of pancreatic alpha cell function.

Both type 1 and type 2 diabetes patients frequently manifest inappropriate glucagon secretion that contributes to the deterioration of glycemic control (1, 2, 3). Recent efforts have focused on modulating the glucagon receptor in an attempt to limit glucagon secretion to maintain normoglycemia. For example, knockout of the glucagon receptor or using a monoclonal antibody against the glucagon receptor have been reported to improve hyperglycemia in an insulin-independent manner (4, 5). Other studies report drugs targeting the glucagon receptor, and glucagon receptor antagonists, as potential approaches to treat diabetes (6, 7). Thus, understanding the mechanism(s) underlying alpha cell function and growth continue to be areas of scientific interest with therapeutic implications.

Several metabolites (8), hormones, and intra-islet factors have been reported to regulate alpha cell function including glucose, somatostatin (9, 10), γ-amino butyric acid (11, 12), Zinc ions (13, 14), acetylcholine (15, 16), and insulin (17, 18, 19, 20). We previously provided genetic evidence for a role for insulin by demonstrating that alpha cell-specific insulin receptor knockout (alphaIRKO) mice exhibit hyperglucagonemia, impaired glucose tolerance, and a lack of suppression of glucagon release in response to insulin stimulation (20). An independent study reported that insulin suppressed preproglucagon gene expression through the nuclear exclusion of Forkhead/winged helix box gene, group O-1 (21). We have also reported that X-box binding protein-1 activates the insulin signaling pathway in alpha cells to regulate glucagon secretion in response to glucose (22). This is consistent with cross-talk between the insulin/IGF-1 signaling and ER stress signaling pathways reported in other cell types (23, 24). However, the relative roles of downstream signaling proteins that are modulated by activating the insulin receptor in alpha cells are not fully elucidated.

It is well established from studies in diverse metabolic cells that signaling at the insulin receptor leads to activation of phosphatidyl 3-kinase and AKT *via* adaptor proteins such as insulin receptor substrate 1 (IRS1) or 2 (IRS2) (25). For example, in pancreatic beta cells, IRS1 and IRS2 play distinct roles in insulin secretion, proliferation, and survival against ER stress (26, 27, 28, 29, 30). To investigate whether these substrates play differential roles in regulating alpha cell biology, we generated alpha cell-specific IRS1 knockout (alphaIRS1KO) and alpha cell-specific IRS2 knockout (alphaIRS2KO) mice for *in vivo* studies and derived stable IRS1 knockdown (alphaIRS1KD) or IRS2 knockdown (alphaIRS2KD) alpha cell lines for *in vitro* experiments. The alphaIRS1KO mice showed impaired glucose tolerance, insulin resistance, and inadequate suppression of glucagon secretion following glucose administration. These findings were complemented by defects in alphaIRS1KD cells *in vitro* with altered translation of glucagon, inadequate Ca^2+^ oscillations, and mitochondrial dysfunction in comparison to control or alphaIRS2KD cells. Together, the data support a prominent role for IRS1 in the regulation of alpha cell secretory function.

## Results

### Alpha cell-specific deletion of IRS1, but not IRS2, leads to glucose intolerance due to dysregulated glucagon secretion

We used alphaIRS1KO, alphaIRS2KO, and corresponding floxed littermate controls that lack Cre for experiments. Glucagon-Cre mice did not show significant differences in glucose tolerance test compared with control (WT) mice (Fig. S1). Furthermore, the lack of phenotypic differences between WT mice and floxed animals in previous studies in our lab (unpublished observations) prompted us to use the floxed mice as controls.

**Figure 1:**
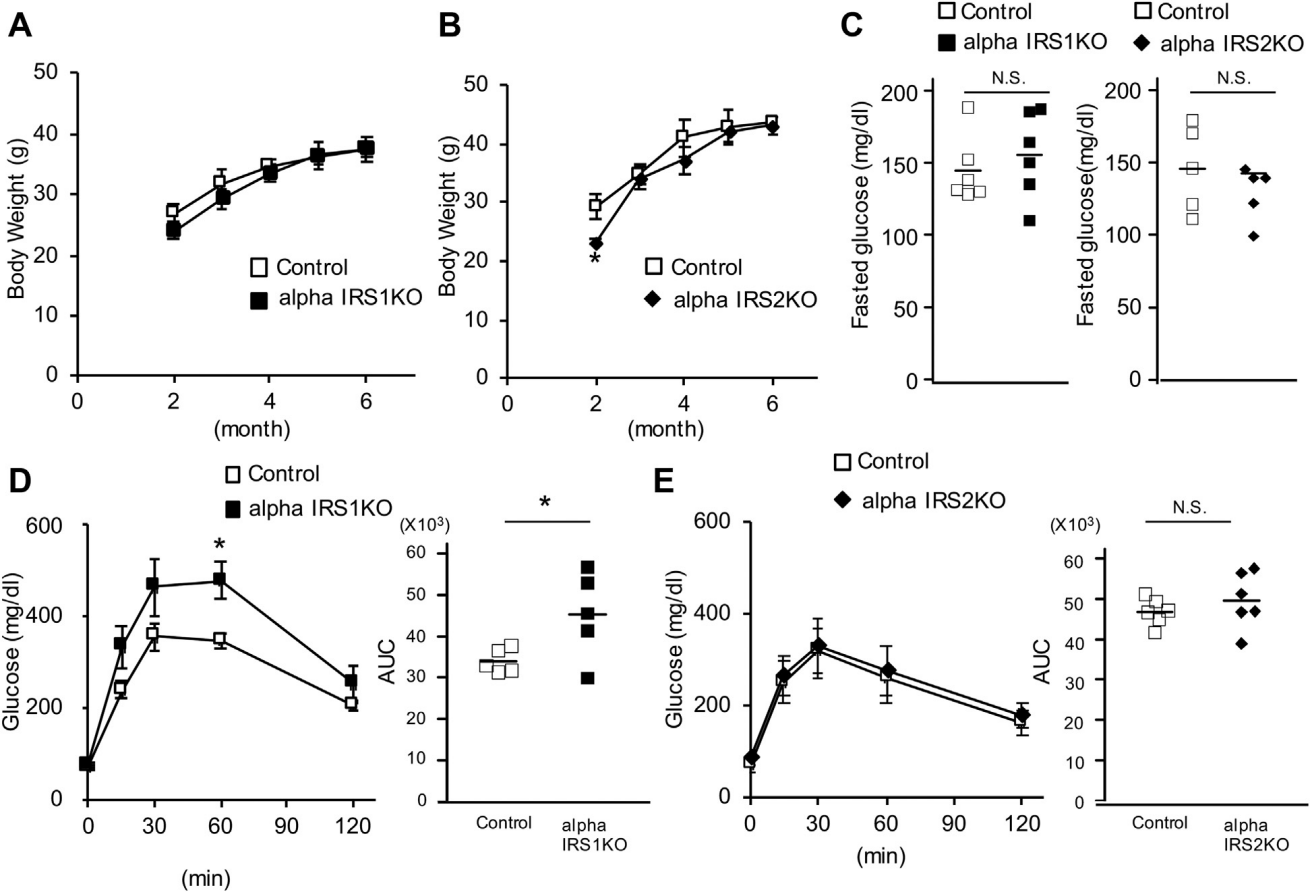

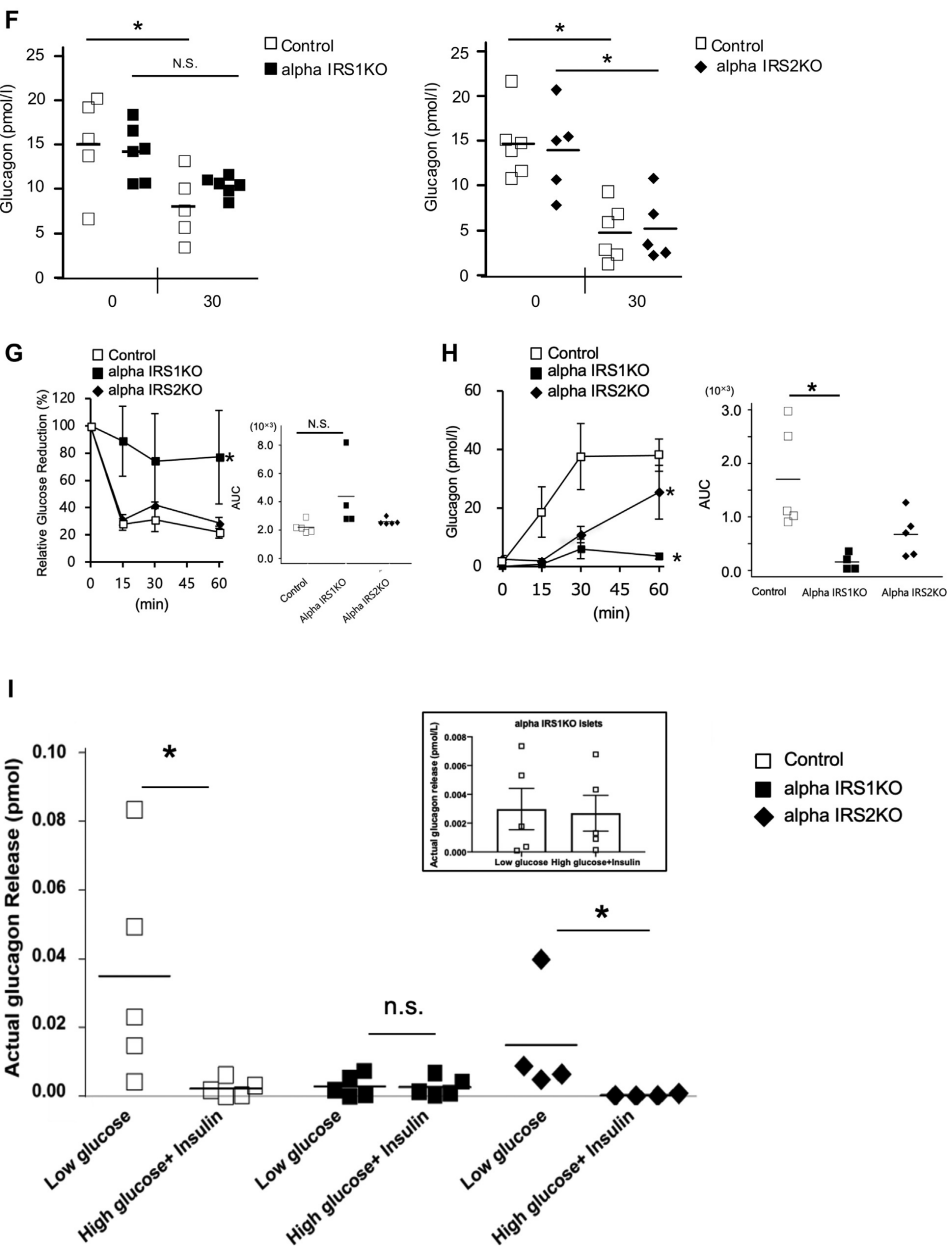
**Mice with alpha cell specific deletion of IRS1 exhibit glucose intolerance due to insufficient glucagon secretion.***A*, body weights of alphaIRS1KO (n = 3–5). *B*, alphaIRS2KO (n = 4). *C*, nonfasting blood glucose in 6-month-old of male alphaIRS1KO (n = 6) and alphaIRS2KO (n = 5). *D*, oral glucose tolerance test (OGTT; glucose 1 g/kg body weight [BW]) in 6-month-old male alphaIRS1KO (n = 5) or *E*, alphaIRS2KO mice (n = 6). In *D* and *E*, quantification is shown in the *right panel*. *F*, serum glucagon levels of OGTT measured by RIA in 6-month-old alphaIRS1KO (n = 5) or alphaIRS2KO (n = 6) mice. *A*–*F*, were analyzed with an unpaired two-tailed Student *t* test. *G* and *H*, plasma glucose (*G*) and glucagon levels (*H*) measured by ELISA following the intraperitoneal injection of insulin (insulin 1 unit/kg BW) to control (n = 5), alphaIRS1KO (n = 4), alphaIRS2KO (n = 5). One-way ANOVA for area under the curve of plasma glucose, F = 3.07, *p* = 0.0874. One-way ANOVA for area under the curve of glucagon, F = 7.02, *p* = 0.0108. Tukey test was applied as a post hoc test. Data indicates the % reduction in glucose. Data are expressed as means ± SD, ∗*p* < 0.05. *I*, glucagon secretion assay of isolated islets. Glucagon secretion data are shown as actual glucagon release in total media (pmol). *Inset* shows the result of alphaIRS1KO. alphaIRS1KO, alpha cell-specific IRS1-knockout; lphaIRS2KO, alpha cell-specific IRS2 knockout; RIA, radioimmunoassay.

All mice were born normally and survived until adulthood, and no significant differences were evident in body weights among groups except for a mild decrease in alphaIRS2KOs at 2 months of age compared with their respective littermate controls (Fig. 1, *A* and *B*). Assessment of glucose homeostasis revealed no significant differences in nonfasting blood glucose levels among groups (Fig. 1*C*). However, in response to oral glucose administration, alphaIRS1KO mice displayed glucose intolerance while blood glucose levels were comparable between control and alphaIRS2KO mice (Fig. 1, *D* and *E*). On the other hand, oral gavage of glucose failed to inhibit glucagon secretion significantly in alphaIRS1KO mice in contrast to the suppression evident in control and alphaIRS2KO mice (Fig. 1*F*). Insulin levels during the oral glucose tolerance test were not different between control and alphaIRS1KO mice (Fig. S2*A*)

To further explore the insulin resistance defects, we examined glucagon responses following insulin injection during the insulin tolerance tests in the two individual knockouts. While a rapid and significant increase in glucagon was evident in controls, the rise was relatively slower in the mutants, and a blunted response was evident in alphaIRS1KO mice (Fig. 1, *G* and *H* and Fig. S2B). The phenotype of insulin resistance in alphaIRS1KO mice could be because of a crosstalk between the target tissue (*e.g.*, alpha cells) and other metabolic organs similar to reports in other tissue-specific models (31) and requires further study.

For further confirmation of a role for IRS1 in insulin-mediated regulation of glucagon secretion and expression, we performed *ex vivo* studies using isolated islets from each genotype of mice. Actual glucagon release and the delta change in glucagon secretion between low glucose and (high glucose + insulin) revealed a blunted inhibitory effect of insulin on glucagon secretion in the islets from alphaIRS1KO mice (Fig. 1*I*). However, when the data were expressed as % of glucagon content (32), the difference did not reach statistical significance between groups. In qRT-PCR, although the suppression of glucagon mRNA by insulin was observed in all groups, the inhibitory effect was significantly smaller in alphaIRS1KO islets compared with control islets (Fig. S3). These results support our hypothesis that IRS1 plays a role in the regulation of glucagon by insulin.

These data suggest that IRS1 plays a major role in regulating glucagon secretion in alpha cells and that hypoglycemia-induced glucagon secretion is linked to insulin sensitivity. Further work is necessary to fully dissect this interaction.

**Figure 2:**
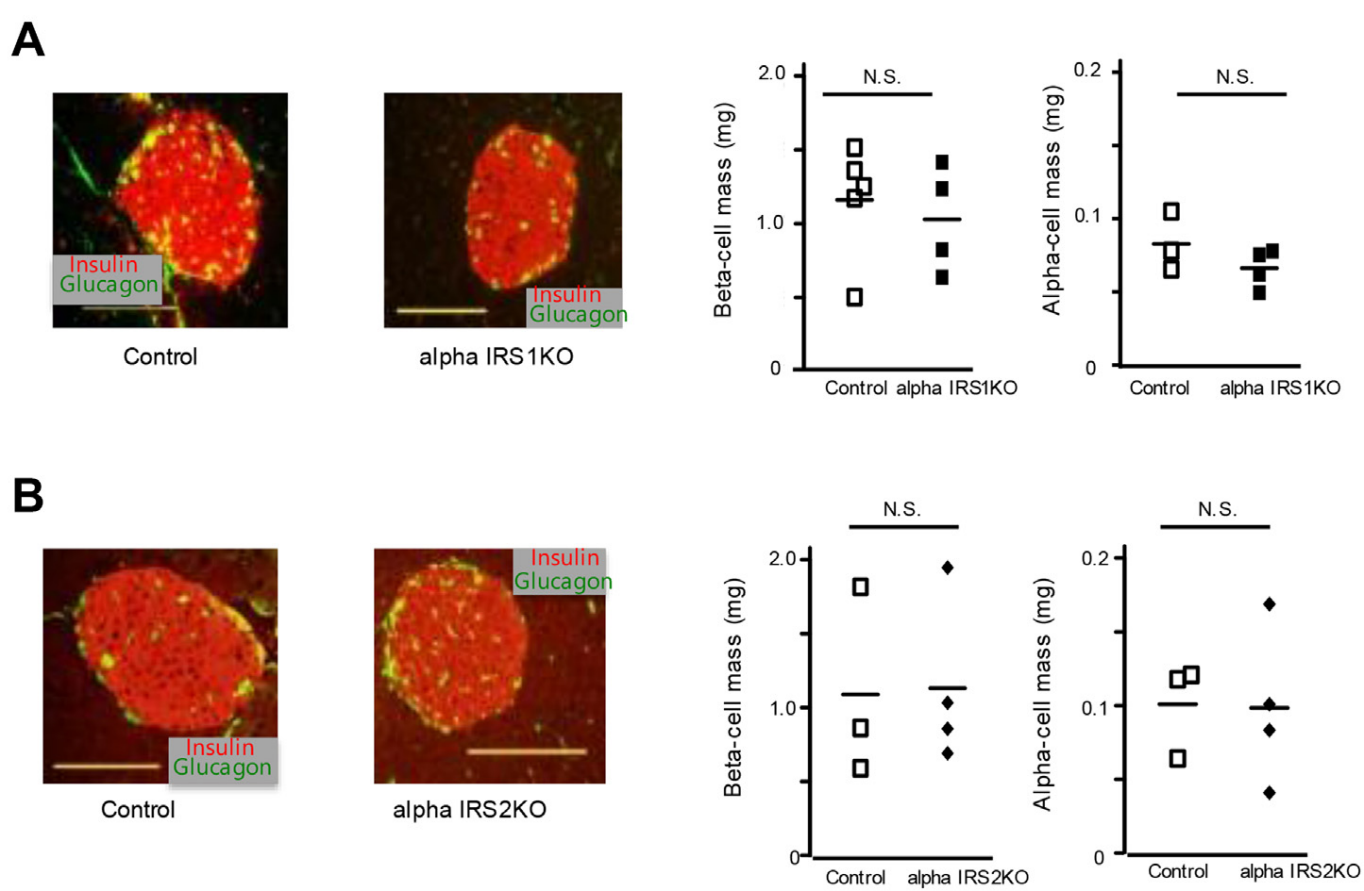
**Alpha cell–specific deletion of IRS1 or IRS2 has no significant effects on alpha or beta-cell mass.***A* and *B*, immunofluorescence staining for insulin (*red*) and glucagon (*green*) in pancreas sections from 6 month old. *A*, alphaIRS1KO or *B*, alphaIRS2KO mice (scale bar, 200 μm). Quantification of beta and alpha cell mass are shown in the *right panels* (n = 4–5), and *lines* are expressed as means. Results were analyzed with an unpaired two-tailed Student *t* test. alphaIRS1KO, alpha cell-specific IRS1-knockout; lphaIRS2KO, alpha cell-specific IRS2 knockout; IRS1, insulin receptor substrate 1; IRS2, insulin receptor substrate 2.

The architecture of the endocrine pancreas and the mass of alpha or beta cells in islets were not significantly different among groups at 6 months of age (Fig. 2, *A* and *B*). Consistently, the proliferation and apoptosis of alpha cells, which were measured by Ki-67 and TUNEL immunostaining respectively, were comparable among groups (Fig. S4, *A* and *B*).

### AlphaIRS1KD and alphaIRS2KD cells exhibit similar blunting in insulin-stimulated AKT phosphorylation

**Figure 3:**
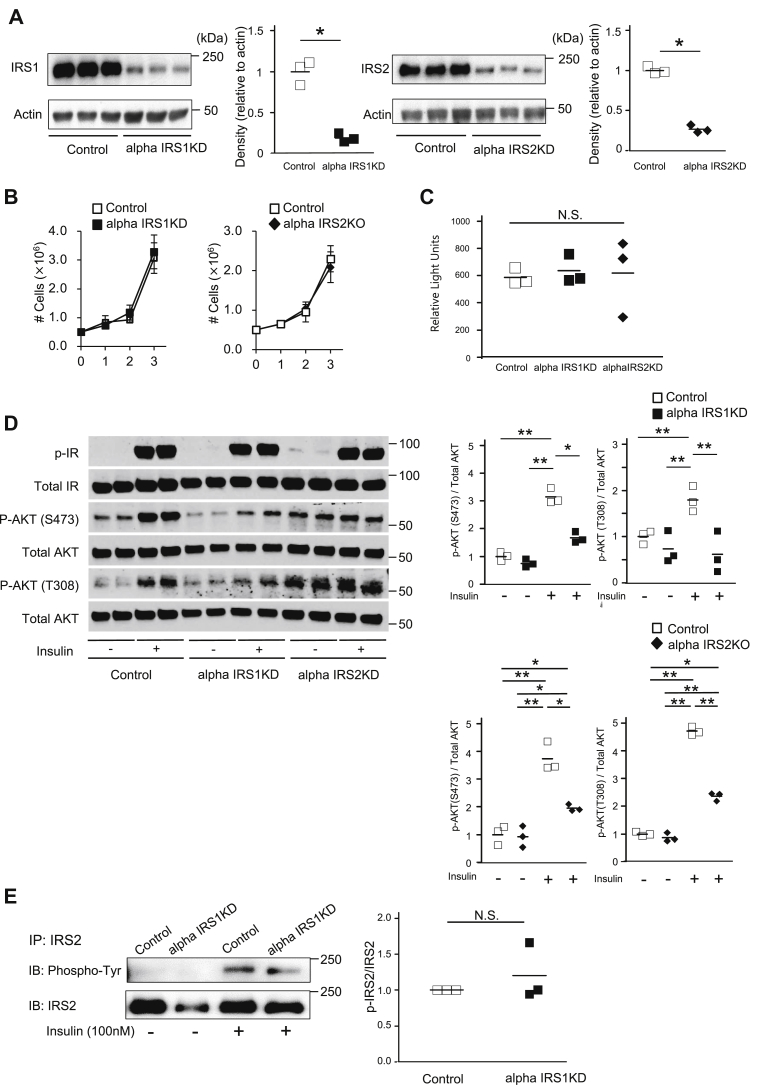
**Deficiency of IRS1 or IRS2 leads to impaired insulin signaling in alpha cells.***A*, Western blotting for IRS1, IRS2, or beta-actin proteins (loading control). *Lines* are expressed as means (n = 3). Quantification is shown in the *right panel*. *B*, control, alphaIRS1KD or alphaIRS1KD cells were plated onto 6-well plates (5 × 10^5^/well), and cells were counted on the indicated day. Data are expressed as means ± SD (n = 3). *C*, BrdU incorporation was measured in control, alphaIRS1KD, or alphaIRS2KD. *Lines* are expressed as means (n = 3). The quantification of BrdU incorporated into cells was measured by relative light units (RLU) using luminometer. *A*–*C*, were analyzed with an unpaired two-tailed Student *t* test. *D*, Western blotting for insulin receptor (IR) tyrosine ^972^ phosphorylation (37), IR, AKT Thr^308^ phosphorylation, AKT Ser^473^ phosphorylation, total AKT protein in control, alphaIRS1KD, and alphaIRS2KD cell lines with or without insulin (100 nM) treatment. *Lines* are expressed as means (n = 3). In control *versus* alpha IRS1KD, AKT S473: Two-way ANOVA, F = 50.88, *p* < 0.0001(IRS1), F = 56.31, *p* < 0.0001(Insulin), F = 72.70, *p* < 0.0001 (Interaction). AKT T308: Two-way ANOVA, F = 10.945, *p* = 0.0107(IRS1), F = 14.463, *p* = 0.0052 (Insulin), F = 7.729, *p* = 0.0239 (Interaction). Tukey test was applied as a post hoc test. In control *versus* alpha IRS2KD, AKT S473: Two-way ANOVA, F = 8.523, *p* = 0.0193(IRS2), F = 62.102, *p* < 0.0001(Insulin), F = 5.060, *p* = 0.0546 (Interaction). AKT T308: Two-way ANOVA, F = 48.24, *p* = 0.0001(IRS2), F = 169.83, *p* < 0.0001(Insulin), F = 12.13, *p* = 0.0082 (Interaction) Tukey test was applied as a post hoc test. Data are expressed as means ± SD (n = 3) with or without insulin (100 nM) treatment. *E*, Western blotting for tyrosine phosphorylation (Tyr), IRS2 in alphaIRS1KD cells after immunoprecipitation with IRS2 antibody. *Lines* are expressed as means (n = 3 in each group). ∗*p* < 0.05, ∗∗*p* < 0.01, N.S. not significant. In *D* and *E*, quantification is shown in the *right panel*. alphaIRS1KD, IRS1 knockdown; alphaIRS2KD, IRS2 knockdown; BrdU, bromodeoxyuridine; IRS1, insulin receptor substrate 1; IRS2, insulin receptor substrate 2.

To clarify the role of IRS1 *versus* IRS2 in intracellular signaling in alpha cells, we generated stable independent knockdowns of IRS1 or IRS2 proteins in alpha TC6 alpha cell lines using lentiviral short hairpin RNAs. Western blotting confirmed ∼80% knockdown of IRS1 or IRS2 protein in the respective cell lines (Fig. 3*A*). Neither alphaIRS1KD nor alphaIRS2KD showed significant changes in cell proliferation as assessed by cell counting or bromodeoxyuridine (BrdU) incorporation assays (Fig. 3, *B* and *C*). To assess the relative contributions of IRS1 *versus* IRS2 in modulating receptor-mediated insulin signaling proteins, we stimulated cells with insulin and observed that AKT phosphorylation (at Ser473 and Thr308 residues) was significantly decreased in both knockdown cell lines to a similar extent (Fig. 3*D*). Interestingly, we did not detect compensatory changes in the expression of either substrate protein in response to stimulation with insulin when one of them was knocked down. The lack of a commercially available high sensitivity pIRS antibody, prompted us to use an immunoprecipitation approach to evaluate the phosphorylation of IRS2 with a phosphotyrosine antibody (Fig. 3*E*). Finally, to directly evaluate the relevance of IRS1 in mediating the signaling effects downstream, we generated IRS1 overexpressing alpha cells (alphaIRS1OE) using adenovirus (33) and subjected them to insulin stimulation. Indeed, alphaIRS1OE cells exhibited an increase in Ser473 phosphorylation of AKT compared with controls (Fig. S5*A*). Consistent with a role for mTOR signaling in the regulation of alpha cell mass and glucagon secretion (34), we observed reduced phosphorylation of S6K in both cell lines after treatment with insulin (Fig. S5*B*).

### AlphaIRS1KD cells exhibit altered glucose-stimulated glucagon mRNA expression and translation to protein

**Figure 4:**
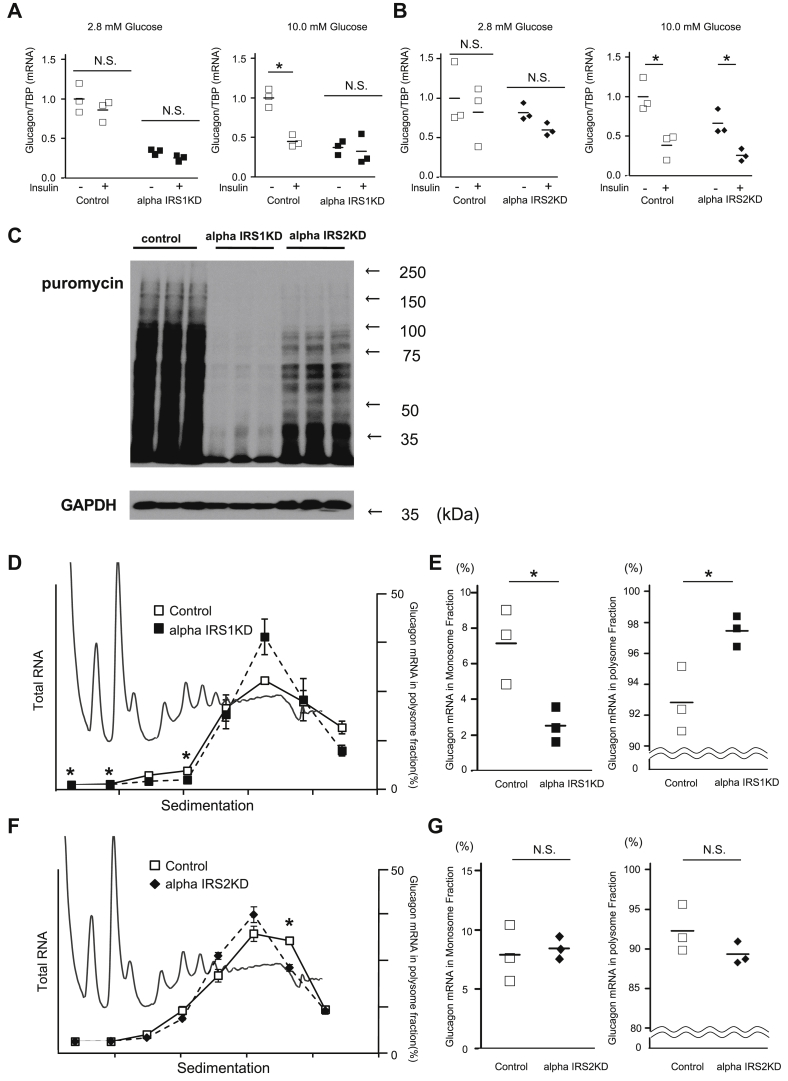
**IRS1 deficiency in alpha cells alters glucagon mRNA expression and translation.***A* and *B*, real-time PCR for glucagon gene expression in (*A*) alphaIRS1KD cells or (*B*) alphaIRS2KD cells. Cells were incubated at 2.8 or 10 mmol/l glucose with/without 100 nmol/l insulin (Ins) for 6 h. *C*, global protein translation was altered in alphaIRS1KD cells. Puromycin incorporation assay in control, alphaIRS1KD, and alphaIRS2KD alpha cells (n = 3). *D*–*G*, polysome profiles for alphaIRS1KD or alphaIRS2KD cells. The difference in the P/M ratio between control and alphaIRS1KD or alphaIRS2KD. P/M indicates ratio of polysomal to monosomal (40S, 60S, and 80S) fractions. Data are means ± SD, n = 3. Glucagon mRNA was quantified in each fraction of sucrose gradient of both control and (*D*) alphaIRS1KD or (*F*) alphaIRS2KD. Glucagon mRNA in monosome and polysome fractions in (*E*) alphaIRS1KD or (*G*) alphaIRS2KD cells was calculated. *Lines* are means, n = 3. ∗*p*-value < 0.05. N.S. not significant. *A*, *B*, and *D*–*G*, were analyzed with an unpaired two-tailed Student *t* test. alphaIRS1KD, IRS1 knockdown; alphaIRS2KD, IRS2 knockdown.

Next, to examine dynamic changes in gene expression, we measured glucagon mRNA in the cell lines in the presence of high or low glucose levels with or without insulin stimulation. Although glucagon mRNA was significantly reduced in response to insulin stimulation at high glucose levels in control and alphaIRS2KD cells, the suppression was not evident in alphaIRS1KD cells even in the presence of insulin (Fig. 4, *A* and *B*), suggesting that IRS1, but not IRS2, mediates the suppressive effects of insulin in alpha cells. We next considered our previous reports that insulin receptor–mediated insulin signaling and IRS1 are involved in global protein translation in beta cells by using polyribosomal profiling (28, 35). To determine which substrate protein contributes to changes in global protein expression in alpha cells, we compared polyribosome profiles between the two knockdown cell lines. A puromycin incorporation assay revealed a reduction in global protein synthesis in alphaIRS1KD cells (Fig. 4*C*). Consistently, islets isolated from alphaIRS1KO mice, but not alphaIRS2KOs, showed a tendency to reduced glucagon content compared to control mice (Fig. S6). To specifically confirm the translation status of glucagon, we collected mRNA from each fraction during polyribosomal profiling gradient analyses and quantified its distribution. Glucagon mRNA in alphaIRS1KD cells was significantly decreased in monosomes and significantly increased in polysomes compared with controls (Fig. 4, *D* and *E*), whereas no difference in expression level in glucagon mRNA was observed between control and alphaIRS2KD cells (Fig. 4, *F* and *G*). Conversely, alphaIRS1OE cells showed a tendency to reduce glucagon mRNA expression in response to insulin stimulation (Fig. S5*C*). These results suggest IRS1 mediates effects of insulin that significantly impact translation, and especially on glucagon protein, in alpha cells.

### AlphaIRS1KD cells display impaired Ca^2+^ oscillations in response to insulin

Alterations in Ca^2+^ flux during the secretory process are a common feature of most secretory cell types (36), and we have previously reported that IRS1-mediated Ca^2+^ flux is involved in insulin secretion (29). To clarify the relative roles of IRS1 *versus* IRS2 in the regulation of Ca^2+^ flux in alpha cells, we measured Ca^2+^ oscillations in cytoplasmic fractions in the presence of low or high glucose levels during insulin stimulation. As expected, and consistent with a suppressive effect of insulin, control alpha cells showed a significant reduction in the number of Ca^2+^ spikes when exposed to high glucose levels in the presence of insulin compared with low glucose.

**Figure 5:**
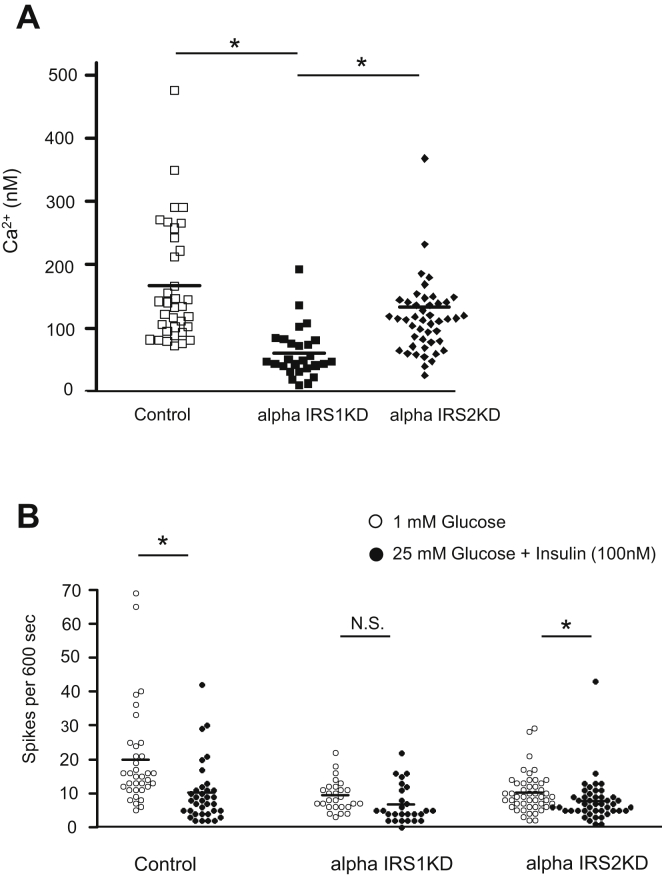
**The effects of IRS1 or IRS2 knockdown on Ca**^**2+**^**-influx in alpha cells.** The glucose concentration was increased from 1 to 25 mmol/l and 100 nmol/l human insulin was added. *A*, the Ca^2+^ concentration in cytosol of each cell at 1 mM glucose is shown (n = 27–47). One-way ANOVA, F = 7.90, *p* = 0.0006. Tukey test was applied as a post hoc test. *B*, quantification of frequency of spikes from each group is shown (n = 27). *Lines* are expressed as means. ∗*p* < 0.05; IRS1, insulin receptor substrate 1; IRS2, insulin receptor substrate 2; N.S., not significant.

In alphaIRS1KD cells, the Ca^2+^ concentration and the frequency of Ca^2+^ spikes were significantly decreased (Fig. 5*A*, Fig. S7, *A* and *B*). In addition, the alphaIRS1KD cells exhibited poor suppression of Ca^2+^ spikes in response to stimulation with high glucose or insulin (Fig. 5*B*). In contrast, the Ca^2+^ spike response in alphaIRS2KD cells was similar to control cells (Fig. 5*B*). These data of a significant decrease in the frequency of Ca^2+^ spikes in alphaIRS1KD cells suggests that absence of IRS1 is linked with altered Ca^2+^ oscillatory response to glucose and insulin stimulation which is not apparent in the IRS2-deficient alpha cells.

### Gene expression microarray analysis of control, alphaIRS1KD, and alphaIRS2KD cell lines

**Figure 6:**
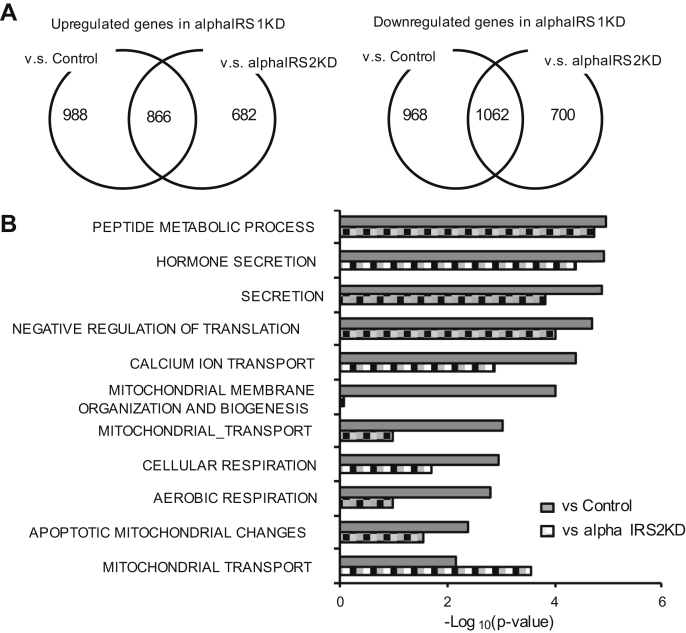

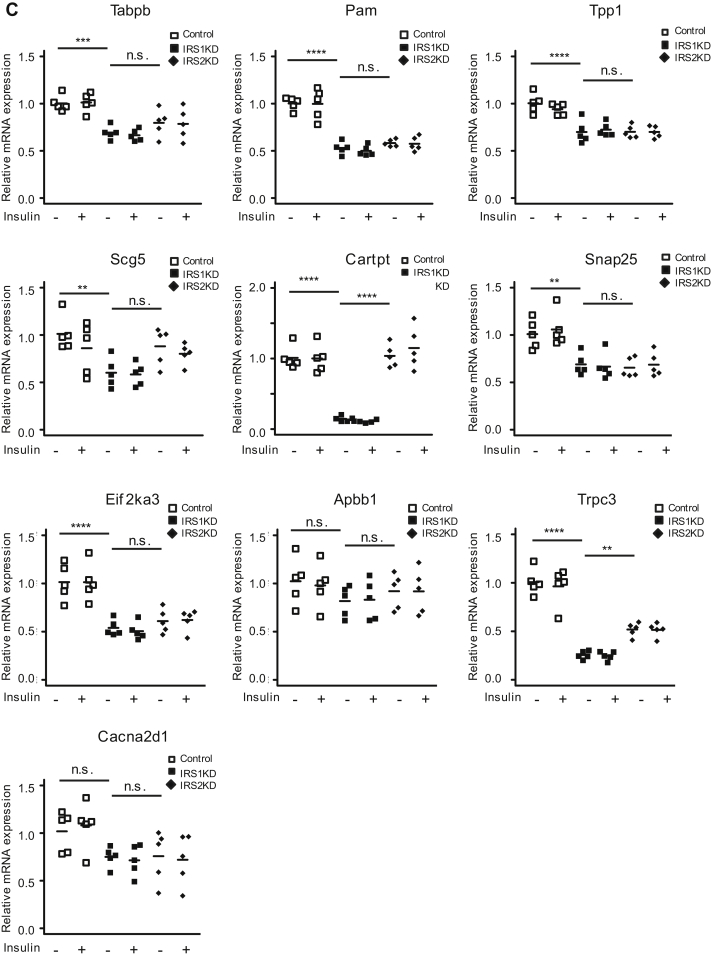
**Microarray analysis of control, alphaIRS1KD, or alphaIRS2KD cells.***A*, shows the number of genes increased/decreased in alphaIRS1KD compared with control and/or alphaIRS2KD cells. *B*, functional analysis of biological pathways that were significantly affected by knockdown. of IRS-1 in alpha cells compared with control or alphaIRS2KD cells (n = 3). *C*, real-time PCR for gene expression in control, alphaIRS1KD, and alphaIRS2KD cells with or without insulin stimulation (n = 5). Cells were starved for 16 h, then stimulated with 100 nM insulin for 6 h. Two-way ANOVA with post hoc Tukey’s test was applied. *Tapbp*: Two-way ANOVA, F = 9.896, *p* = 0.0041(IRS), F = 0.032, *p* = 0.8602 (Insulin), F = 0.021, *p* = 0.8856 (Interaction), *Pam*: Two-way ANOVA, F = 33.816, *p* < 0.0001(IRS), F = 0.060, *p* = 0.809 (Insulin), F = 0.001, *p* = 0.981 (Interaction), *Tpp1*: Two-way ANOVA, F = 34.831, *p* < 0.0001(IRS), F = 0.127, *p* = 0.725(Insulin), F = 0.450, *p* = 0.508 (Interaction), *Scg5*: Two-way ANOVA, F = 0.850, *p* = 0.365(IRS), F = 0.968, *p* = 0.334(Insulin), F = 0.124, *p* = 0.728 (Interaction), *Cartpt*: Two-way ANOVA, F = 0.156, *p* = 0.696(IRS), F = 0.015, *p* = 0.903(Insulin), F = 0.073, *p* = 0.789 (Interaction), *Snap25*: Two-way ANOVA, F = 25.571, *p* < 0.0001(IRS), F = 0.106, *p* = 0.748(Insulin), F = 0.015, *p* = 0.905 (Interaction), *Eif2ak3*: Two-way ANOVA, F = 19.988, *p* = 0.0001(IRS), F = 0.011, *p* = 0.916 (Insulin), F = 0.005, *p* = 0.944 (Interaction), *Apbb1*: Two-way ANOVA, F = 0.750, *p* = 0.005(IRS), F = 0.018, *p* = 0.893(Insulin), F = 0.049, *p* = 0.827 (Interaction), *Trpc3*: Two-way ANOVA, F = 14.723, *p* = 0.0007 (IRS), F = 0.035, *p* = 0.852 (Insulin), F = 0.027, *p* = 0.871 (Interaction), *Cacna2d1*: Two-way ANOVA, F = 9.558, *p* = 0.0047 (IRS), F = 0.002, *p* = 0.962 (Insulin), F = 0.246, *p* = 0.624 (Interaction), n.s. not significant, ∗*p* ≤ 0.05, ∗∗*p* ≤ 0.01, ∗∗∗*p* ≤ 0.001, ∗∗∗∗*p* ≤ 0.0001. alphaIRS1KD, IRS1 knockdown; alphaIRS2KD, IRS2 knockdown.

Results in Figure 3, *D* and *E* indicate that both alphaIRS1KD and alphaIRS2KD cells showed a similar level of reduction in AKT phosphorylation upon stimulation with insulin. One interpretation of these data is that signaling pathways independent of AKT activation are involved in the dysregulation of glucagon secretion in alphaIRS1KD alpha cells. To identify candidate genes and identify pathways that are involved in the regulation of IRS1-mediated signaling in alpha cells, we performed gene expression microarray analysis of control, alphaIRS1KD, or alphaIRS2KD alpha cells. Principal component analysis revealed gene expression patterns for control, alphaIRS1KD, and alphaIRS2KD cells that allowed them to be categorized individually (Fig. S8*A*), suggesting that IRS1 and IRS2 each distinctly regulates the transcriptome of alpha cells. To explore the signaling pathway of IRS1-mediated regulation of glucagon secretion in alpha cells, we investigated genes that are commonly modified in alphaIRS1KD compared with genes in the other groups. We detected 866 genes to be significantly increased, whereas 1062 genes were significantly decreased in alphaIRS1KD compared with both control and alphaIRS2KD groups (Fig. 6*A*, left and right panels). Gene set enrichment analyses (Broad Institute) of significantly altered genes in alphaIRS1KD showed the top pathways included peptide metabolic processes, hormone secretion, translation, calcium ion transportation, mitochondrial organization, and mitochondrial function (Fig. 6*B*). To confirm these findings, we stimulated control, alpha IRS1KD, or alpha IRS2KD cells with 100 nM insulin and examined the expression of genes involved in the pathways mentioned above by qRT-PCR (Fig. 6*C*). We examined genes that are involved in metabolic processes (*e.g.*, *Tapbp*, *Pam*, *Tpp1*), hormone secretion (*e.g.*, *Scg5*, *Cartpt*, *Snap25*), negative regulation of translation (*e.g.*, *Eif2ak3*, *Apbb1*), or calcium ion transport (*e.g.*, *Trpc3*, *Cacna2d1*). In alpha IRS1KD cells, *Cartpt* in the hormone secretion pathway and *Trpc3* in the calcium ion transport pathway were significantly decreased compared with control or alpha IRS2KD cells. None of the other genes examined were significantly altered in alpha IRS1KD cells compared with control or alpha IRS2KD cells. Expression of Ano1/TMEM16A, which is a Ca^2+^-activated Cl^−^ channel, was also increased in alphaIRS1KD cells compared with control or alphaIRS2KD cells (Fig. S8, *B* and *C*). Pathway analysis revealed that “cellular response to stress” was increased in alphaIRS1KD compared with control and alphaIRS2KD, and “negative regulation of phosphorylation and phosphate metabolic process” was upregulated in alphaIRS1KD cells (Fig. S9). These data support the notion that IRS1 has distinct roles in modulating Ca^2+^ oscillations in alpha cells.

To define potential mechanisms that regulate glucagon mRNA expression downstream of the IR-IRS1 axis, we analyzed transcription factor binding sites to identify significantly upregulated or downregulated (<50%) genes within 1 kbp of the promoter region of the glucagon gene. We identified Foxp2, Hoxd13, and Foxj2 as candidate factors for regulation of glucagon transcription through IRS1. Further analyses of the microarray data pointed to alternative mechanisms that are regulated by IRS1 including genes linked with the mTOR, S6K, and 4EBP families. The selected pathways and representative transcription factors associated with these candidates are shown in Fig. S10 and include CXCR3, NFAT, angiopoietin receptor, and RXR–VDR pathways. Analysis of these candidate transcription factors is warranted to investigate their relevance in mediating the direct effects of IRS1 on alpha-cell biology.

### Mitochondrial function is suppressed in alphaIRS1KD cells

**Figure 7:**
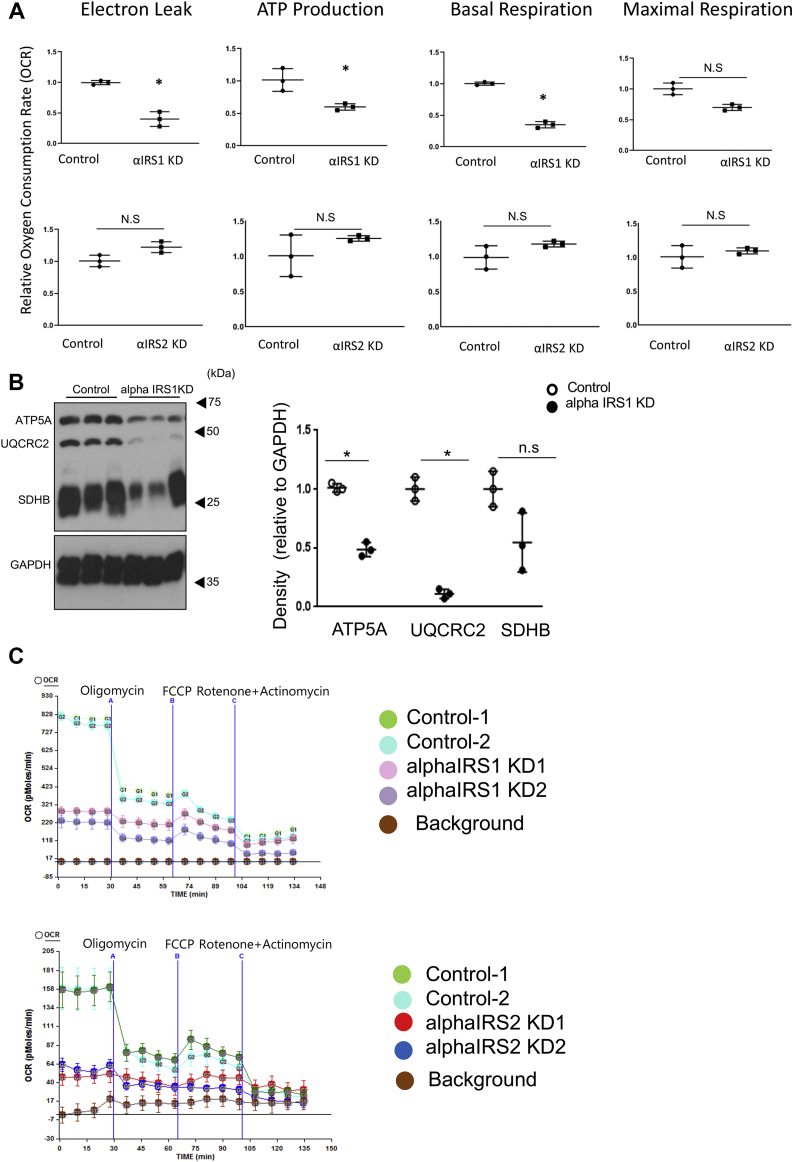
**IRS1 deficiency suppresses mitochondrial function in alpha cells.***A*, electron leak, ATP synthesis, basal respiration, and maximal respiration displayed as a ratio of oxygen consumption rate between control and alphaIRS1KD cells or alphaIRS2KD cells (n = 5–10), measured by the Seahorse XF24 analyzer. *B*, Western blotting for oxidative phosphorylation (OXPHOS) proteins, including ATP synthase subunit alpha (ATP5A, complex V), ubiquinol-cytochrome c reductase core protein II (UQCR2, Complex III), and succinate dehydrogenase iron-sulfur subunit, mitochondrial (SDHB, complex II) in control and alphaIRS1KD cells. (n = 3). Data are expressed as. means ± SD. ∗*p* < 0.05. *A* and *B*, were analyzed with an unpaired two-tailed Student *t* test. *C*, sea horse analyses of control, alphaIRS1KD cells, and alphaIRS2KD cells. alphaIRS1KD, IRS1 knockdown; alphaIRS2KD, IRS2 knockdown; IRS1, insulin receptor substrate 1.

In addition to the changes in Ca^2+^ dynamics, bioinformatics analysis of microarrays revealed alterations in pathways that modulate mitochondrial function (Fig. 6*B*). This observation gains significance in the context of reports that insulin and other growth hormone signaling pathways directly impact cellular biology by regulating mitochondrial function (37, 38). Indeed, evaluation of mitochondrial function in alphaIRS1KD cells showed reduced electron leak, lower ATP synthesis, and blunted basal or maximal respiration compared with control cells (Fig. 7*A*, upper panels, and 7C). In contrast, alphaIRS2KD cells showed no significant differences in these mitochondrial function parameters compared with controls (Fig. 7, *A* and *C*). Western blotting confirmed the reduced expression of the mitochondrial oxidative phosphorylation (OxPhos) proteins, namely ATP synthase subunit alpha (ATP5A, complex V) and ubiquinol-cytochrome c reductase core protein II (UQCR2, Complex III) in alphaIRS1KD cells (Fig. 7*B*).

## Discussion

The factors that regulate alpha cell biology continue to be of great scientific interest to allow better therapeutic targeting in diabetes. We have previously provided direct genetic evidence to implicate insulin action in alpha cells to suppress glucagon secretion *in vivo* (20, 22) consistent with studies reporting a role for insulin in regulating alpha cell biology *in vivo* [reviewed in (39)]. Although blockade of IRS1 in rat “whole” islets leads to impaired glucose-induced lowering of glucagon secretion (40), the presence of other cell types in whole islets does not exclude mechanisms that are independent of IRS1 in alpha cells (41). However, our study using complementary *in vitro*, *in vivo*, and *ex vivo* models circumvents these limitations and points to a direct role of IRS1 in the regulation of glucagon secretion. We report that IRS1 and IRS2, critical downstream proteins in the insulin/IGF-1 signaling pathway, exhibit distinct roles in regulating alpha cell biology. AlphaIRS1KOs partially recapitulated the phenotype of alpha cell-specific insulin receptor knockout (alphaIRKO) mice by exhibiting impaired glucose tolerance due to altered glucagon secretion after glucose administration (20). Because fasting or high fat diet can also modify the kinetics of glucagon *in vivo* (42), a decline in glucagon clearance in alphaIRS1KO may contribute to a delay in the suppression of circulating glucagon levels. Interestingly, neither alphaIRS1KO nor alphaIRS2KO mice showed significant alterations in alpha cell or beta cell areas compared with controls at 6 months of age. The lack of significant differences in proliferation indicate that the insulin receptor substrates are unlikely to directly regulate alpha cell proliferation in contrast to their role in β-cells (43, 44). It is notable that glucagon receptor KO mice demonstrate a significant increase in alpha cell mass (4), pointing to a role for glucagon receptor signaling in alpha cell proliferation.

We have previously reported that insulin negatively regulates glucagon gene transcription (20). In the current manuscript, the *in vitro* model (*i.e.*, alpha TC6 cells) showed that a lack of IRS1, but not IRS2, alters glucagon transcription in response to exogenous insulin. In the *ex vivo* model (*i.e.*, isolated mouse islets) islets from alphaIRS1KO mice showed a significantly smaller suppression than control islets. Further studies are warranted to investigate the molecular mechanisms of transcriptional regulation in the latter which may be differentially impacted by intra-islet paracrine regulation and/or systemic factors. Because the decrease in glucagon secretion was observed in the absence of IRS1, we also studied the expression level of prohormone convertase 2 (PC2) which is involved in processing proglucagon into glucagon. As shown in Fig. S2, there was no change in PC2 expression between control and alpha IRS1KO mice, suggesting that an impaired proglucagon processing is not the dominant cause of the phenotype. In contrast to a role for AKT phosphorylation in the suppression of glucagon in IRKO alpha cells (20, 45), we noted that both alphaIRS1KD and alphaIRS2KD cells showed reduced phosphorylation of AKT. We also observed a reduced phosphorylation of S6K in both cell lines. These results indicate that the two substrate proteins are linked to different signaling proteins that can in turn activate downstream effects. For example, in addition to the AKT and/or mTOR signaling pathway, the PASK/AMPKalpha2 pathway can also independently participate in insulin receptor substrate-mediated regulation of glucagon release (46). Meanwhile, the marked decrease in glucagon secretion, glucagon gene expression, mitochondrial function, as well as *Trpc3* and *Cartpt* gene expression in alphaIRS1KD cells even in the absence of insulin point to an insulin-independent pathway and requires a careful assessment of the ligands that can regulate this insulin receptor substrate.

The reduced glucagon expression observed in the basal state in alphaIRS1KD cells was similar to the observation in XBP1 knock down alpha cells (alpha XBP1KD). The lack of suppression of glucagon gene expression after insulin stimulation in alpha XBP1KD cells was associated with incomplete exclusion of Forkhead/winged helix box gene, group O-1 from the nucleus (22). The markedly reduced phosphorylation of IRS1 in alpha XBP1KD cells suggests that a pathway common to these two proteins is involved in the regulation of glucagon gene expression and warrants further investigation. In contrast to the findings in alphaIRS1KD cells, alphaIRS2KD cells demonstrated normal glucagon gene expression and secretion. Interestingly, despite normal glucagon secretion at 2 months of age alphaIRS2KO mice displayed slightly reduced body weights, insulin resistance, and reduced AKT phosphorylation in response to insulin. The systemic effects in alphaIRS2KO mice might involve pathways independent of regulation *via* AKT phosphorylation. A recent study reported that liver, muscle, and adipocytes from global IRS1KO mice show altered microRNA expression patterns leading to increased IRS2 transcription (47). Thus, the effects of manipulating IRS2 action in alpha cells on the regulation of whole-body metabolism require further study.

The effect of absent IRS1 on glucagon expression was confirmed by a skewed distribution of glucagon mRNA in alphaIRS1KD cells by polysome profiling. In this context, it is notable that IRS1 forms high molecular mass complexes that include proteins related to regulation of protein translation such as EIF4G, EIF4E, and PABP (48). Because the binding of IRS1 was independent of tyrosine phosphorylation (48), the data suggest that IRS1 itself regulates translation of proteins independent of insulin stimulation. Our data also suggest that translation of glucagon is regulated *via* IRS1, and not IRS2, and is independent of the AKT pathway. One possibility is that a reduction in IRS1 affects the stability of the ribosome complex and subsequently alters translation of global proteins, including the hormone glucagon in alpha cells.

A notable finding is that Ca^2+^ flux was different in alphaIRS1KD cells. In alpha cells, the presence of low glucose concentration induces Ca^2+^ oscillations *via* action potentials mediated by voltage-dependent K^+^ channels. At high glucose concentrations, an increased ATP/ADP ratio blocks the K_ATP_ channel, depolarizes the membrane potential, and inactivates the ion channels involved in action potentials (49). We have previously reported that alpha cells with a knockdown of XBP1, which suppressed insulin signaling *via* the IRE1 alpha JNK pathway, demonstrated dysregulation of Ca^2+^ oscillations (22). This current study implicates IRS1 in suppressing Ca^2+^ oscillations at high glucose concentrations in alpha cells, whereas lack of IRS2 does not directly impact Ca^2+^ oscillations. In beta cells, IRS1/2–phosphatidyl 3-kinase signaling is involved in releasing intracellular Ca^2+^ *via* interactions with the ER (50). The finding that both alphaIRS1KD cells and alphaIRS2 KD cells showed lower Ca^2+^ spikes at 1 mM glucose compared with control cells implies similar signaling pathways are operative in beta cells that affect Ca^2+^ levels in alpha cells. We also reported that a lack of IRS1 in β-cells affected Ca^2+^ flux by reducing sarcoplasmic endoplasmic reticulum Ca^2+^-ATPase (SERCA) expression (29). The absence of changes in SERCA expression in alphaIRS1KD cells in our microarray analysis suggests the alterations in Ca^2+^ flux is likely modulated by channels on the plasma membrane to regulate extracellular Ca^2+^ influx. Considering deletion of the *Trpc3* gene has been reported to reduced Ca influx (51), it is possible that a significant decrease in its expression in alpha IRS1KD cells contributes to the dysregulation of Ca^2+^ oscillations in alpha IRS1KD cells. The alterations in *Cartpt* expression in alpha IRS1KD warrants further studies to dissect the link between the insulin signaling pathway and hormone secretion in alpha cells (52). It will also be worth examining whether the increased expression of Ano1/TMEM16A in alphaIRS1KD cells, as evident in the microarray analyses, is involved in glucagon secretion given their role in glucose stimulated insulin secretion in beta cells (53).

Alpha cell secretory function has been reported to be linked to mitochondrial function (54); however, this association has not been fully investigated in the context of the insulin/IGF-1 signaling pathway. Our studies indicate that alpha cells lacking IRS1 exhibit decreased mitochondrial function including basal respiration rate, maximal respiration rate, and ATP synthesis, that is complemented by a reduction in expression of OXPHOS proteins. Indeed, ATP synthesis is necessary to close K_ATP_ channels regulating alpha cell membrane potential when glucose levels are high (49). Thus, the decreased ATP production in alphaIRS1KD cells could lead to inadequate suppression of glucagon secretion. A previous study has suggested distinct effects of IRS proteins on mitochondrial function in cardiomyocytes (55), and the authors reported that IRS2-kockout cardiomyocytes showed decreased PGC1alpha and imply a tissue-dependent role for IRS proteins in regulating mitochondrial function. A recent report suggested that the change in mitochondrial function in alpha cells can exacerbate glucose homeostasis through uninhibited insulin secretion because of impaired glucagon signaling (56). Knockdown of IRS1 in alpha cells also demonstrated mitochondrial dysfunction in this study, and there continues to be considerable interest in elucidating the significance of mitochondrial dysregulation in alpha cell metabolism.

In summary, we report that IRS1 plays a distinct role in glucagon secretion in alpha cells by modulating protein translation, mitochondrial function, and Ca^2+^ oscillation. These studies suggest that altering proteins in the insulin/IGF1 signaling pathway have implications for regulating glucagon secretion in pathophysiological states.

## Experimental procedures

### Mouse breeding and physiological experiments

All protocols were approved by the Brandeis University Institutional Animal Care and Use Committee. Mice were maintained on a 12-h light/dark cycle at the Foster Biomedical Research Laboratory of the Brandeis University in Waltham, Massachusetts. The IRS1-floxed mice (57), IRS2-floxed mice (58), and glucagon-Cre mice (59, 60) were intercrossed to generate alpha cell-specific IRS1 knockout (alphaIRS1KO) mice and alpha cell-specific IRS2 knockout (alphaIRS2KO) mice. Knockout efficiency of the Gcg-Cre mouse was confirmed in our former publication (22).The alphaIRS1KO, alphaIRS2KO, and their corresponding littermate control floxed mice were maintained on a C57/BL6 and 129 Sv mixed genetic background. Initial studies revealed that male and female mice showed similar phenotypes, and we undertook detailed experiments only in male mice (15–21 weeks old). Blood glucose was measured with a Glucometer (Contour, Bayer). Plasma insulin was monitored by ELISA (Crystal Chem), plasma glucagon by radioimmunoassay (Linco) and ELISA (Mercodia Inc), and plasma glucagon-like-peptide 1 by ELISA (Linco). Glucose and insulin tolerance tests were performed as described previously (20, 22, 27).

### *Ex vivo* studies of isolated islets

Mouse islets were isolated from control, alphaIRS1KO, and alphaIRS2KO mouse with 12 to 15 weeks old according to our previous protocol (22). Ten and forty size-matched islets were used for secretion assay and qRT-PCR, respectively.

For glucagon secretion assay, islets were preincubated in Krebs-Ringer buffer (KRB) supplemented with 3.3 mmol/l glucose for 1 h followed by the incubation in 1 ml KRB with 16.7 mmol/l glucose supplemented with 100 nM insulin for 1 h at 37 °C. Aliquots were stored at −80 °C until the measurement of glucagon. Glucagon was measured by ELISA, as described above. Glucagon secretion was normalized by glucagon content in digested islets.

For qRT-PCR, islets were incubated in KRB buffer with 3.3 mmol/l or 16.7 mmol/l glucose supplemented with or without 100 nM insulin for 24 h at 37 °C. After the incubation, samples were centrifuged in 1500 rpm for 5 min followed by the discard of the aliquots. Pellets were kept frozen in −80 °C until RNA extraction.

### Immunohistological analysis

Mice were anesthetized, and the pancreas was rapidly dissected, fixed with Z-Fix (Anatech), and embedded in paraffin. Paraffin sections were immunostained for insulin (Abcam), glucagon (Sigma-Aldrich), and somatostatin (Abcam). Photomicrographs were obtained with a charge-coupled device camera, and the beta-cell, alpha cell, and total pancreatic areas were estimated using ImageJ software (NIH).

### BrdU incorporation assay

BrdU labeling kits were obtained from Abcam. We measured BrdU incorporation to assess cell proliferation according to the manufacture’s protocol. Briefly, cells were cultured in the presence of BrdU followed by incubation with anti-BrdU antibody after fixation. Cells were incubated with horseradish peroxidase conjugate antibody. Chemiluminescent substrate was added, and luminescence was measured by luminometer. Results were shown in relative light units.

### Cell culture

Alpha TC6 cells were cultured with RPMI 1640 medium with 10% fetal bovine serum, and experiments were performed using 80 to 90% confluent cells. Alpha TC6 cells were infected with a lentivirus having small hairpin RNA (shRNA) for nonsilencing control, IRS1, or IRS2 to generate stable cell lines *via* puromycin selection as previously described (22). Lentiviral vector plasmids for murine IRS1 and IRS2 shRNA and control nonsilencing shRNA were purchased from Open Biosystems.

### SDS-PAGE and Western blotting

Cells were lysed for Western blotting analysis with radioimmunoprecipitation buffer as reported previously (28). Total protein concentration was determined using a bicinchonic acid assay (Pierce). Samples were resuspended in reducing SDS-PAGE sample buffer, boiled, and resolved by SDS-PAGE. Proteins were subsequently transferred onto polyvinylidene difluoride membranes, treated with blocking buffer (Thermo Scientific), and incubated with primary antibodies overnight at 4 °C. Beta-actin, GAPDH, pAKT (pT308), pAKT (pS473), total AKT, and IRS2 antibodies were from Cell Signaling. pIR (pY972) and pIRS1 (pY896) antibodies were from Invitrogen. Phospho-tyrosine antibody was from SantaCruz. IRS1 antibody was from Millipore. Total OXPHOS Rodent WB Antibody Cocktail was from Abcam.

### Immunoprecipitation

Cells were lysed with radioimmunoprecipitation buffer. The lysate was centrifuged at 12,000 rpm for 30 min at 4 °C. Supernatant was collected and rotated overnight with beads (Clontech) at 4 °C. Beads were washed three times with TBS. Proteins were eluted into SDS sample buffer by heating for 10 min at 95 °C. Proteins were resolved on SDS-PAGE and detected by immunoblotting.

### Real-time PCR

RNA was extracted from cells using RNeasy Mini Kit (QIAGEN), and 1 mg RNA was used for a reverse transcription step using the high-capacity cDNA Archive Kit (Applied Biosystems). cDNA was analyzed and amplified using the ABI 7900HT system (Applied Biosystems). TATA-binding protein was used as an internal control. Primers for glucagon: 5′-TGAATTTGAGAGGCATGCTG-3′ and 5′-TGGTGCTCATCTCGTCAGAG-3′. Primers for TATA-binding protein: 5′-ACCCTTCACCAATGACTC CTATG-3′ and 5′-ATGATGACTGCAGCAA ATCGC-3′. Primers for Figure 6*C* is listed in Fig. S11.

### Polyribosomal profiling experiments

Polyribosomal profiling was performed as previously described with modifications (28, 61). Briefly, after incubation with 100 μg/ml cycloheximide (CHX) for 10 min at 37 °C, cells were washed in ice-cold PBS containing CHX (50 μg/ml), and lysed in 300 μl polysome buffer (200 mM KCl, 20 mM Tris HCl [pH 7.4], 10 mM MgCl2, 1% Triton-X, 50 U/ml RNasin [Promega], 100 μg/ml CHX). The cell lysates were homogenized using a 23-gauge needle and incubated on ice for 10 min followed by centrifugation at 13,000*g* for 10 min at 4 C. Supernatant was layered onto a 10 to 50% sucrose gradient solution containing 20 mM Tris HCl (pH 7.4), 10 mM MgCl2, 200 mM KCl, and 50 μg/ml CHX. The sucrose gradients were subjected to centrifugation at 4 °C in a Beckman SW-41Ti rotor at 39,000 rpm for 2 h. A piston gradient fractionator (BioComp Instruments) was used to fractionate the gradients and absorbance of RNA at 254 nm was recorded using an on-line UV monitor.

### Measurements of cytoplasmic Ca^2+^ concentrations

Cells on coverslips were incubated for 40 min with 2 mmol/l Fura-2 acetoxymethyl ester at 37 °C and washed by KRB with 0.5 mmol/l glucose. Cells on coverslips were incubated for 30 min with 2 mol/l Fura-2 acetoxymethyl ester (Molecular Probes) at 37 °C in RPMI 1640 media. Coverslips containing the fura-2 loaded islets or cells were placed in a coverslip holder with a capacity of 300 l, and experiments were performed in KRB.

Excitation of fura-2 was accomplished using an Xe lamp with sequential excitation at 340 and 380 nm. Fluorescence emission was collected and stored using DM3000 M data acquisition software (Instruments SA) for quantification of Ca^2+^ concentration as described earlier (29).

### Oxygen consumption assay and bioenergetics analysis

Cells were seeded in XF 24-well cell culture microplates (Seahorse Bioscience) 1 day before analysis at 5.0 × 10^4^ cells/well. Oxygen consumption of cell lines was analyzed using the XF24 Extracellular Flux Analyzer (Seahorse Bioscience). A bioenergetics including basal mitochondrial respiration, ATP synthesis, electron leak, mitochondrial respiratory capacity, and nonmitochondrial respiration was measured with oligomycin (10 μM), carbonyl cyanide p-trifluoromethoxyphenylhydrazone (1 μM), and rotenone (5 μM) which were sequentially added in wells according to the manufacture’s protocol.

### Microarray

The samples from all the conditions (control, alphaIRS1KD, and alphaIRS2KD) were examined in triplicate. The samples were processed following the Affymetrix GeneChip WT Plus Reagent Kit (cat # 703174) followed by the Affymetrix GeneChip WT Terminal Labeling and Hybridization (cat # 702808) onto Affymetrix Mouse Gene 2.0ST arrays. The arrays were washed and stained on Fluidics Stations 450 using the Affymetrix Hybe/Wash/Stain (cat # 900720). The arrays were scanned on GeneChip Scanner 3000 7G.

### Overexpression model of IRS1

The adenovirus vector expressing IRS1 was kindly provided by G. L. King MD (Joslin Diabetes Center) (33). For infection with adenovirus vector, it was transfected by multiplicity of infection of 5.

### Statistical analysis

All data are expressed as means ± SD and were analyzed with an unpaired two-tailed Student *t* test or ANOVA and post hoc tests. Differences were considered significant at *p* < 0.05.

## Data availability

The microarray data were deposited in Gene Expression Omnibus (accession number: GSE130329). All the other data are contained within this manuscript.

## Supporting information

This article contains supporting information.

## Conflicts of interest

The authors declare that they have no conflicts of interest with the contents of this article.

## References

[1] D'Alessio D. (2011). The role of dysregulated glucagon secretion in type 2 diabetes. Diabetes Obes. Metab..

[2] Unger R.H., Cherrington A.D. (2012). Glucagonocentric restructuring of diabetes: A pathophysiologic and therapeutic makeover. J. Clin. Invest..

[3] Campbell J.E., Drucker D.J. (2015). Islet alpha cells and glucagon--critical regulators of energy homeostasis. Nat. Rev. Endocrinol..

[4] Gelling R.W., Du X.Q., Dichmann D.S., Romer J., Huang H., Cui L., Obici S., Tang B., Holst J.J., Fledelius C., Johansen P.B., Rossetti L., Jelicks L.A., Serup P., Nishimura E. (2003). Lower blood glucose, hyperglucagonemia, and pancreatic alpha cell hyperplasia in glucagon receptor knockout mice. Proc. Natl. Acad. Sci. U. S. A..

[5] Okamoto H., Kim J., Aglione J., Lee J., Cavino K., Na E., Rafique A., Kim J.H., Harp J., Valenzuela D.M., Yancopoulos G.D., Murphy A.J., Gromada J. (2015). Glucagon receptor blockade with a human antibody normalizes blood glucose in diabetic mice and monkeys. Endocrinology.

[6] Kelly R.P., Garhyan P., Raddad E., Fu H., Lim C.N., Prince M.J., Pinaire J.A., Loh M.T., Deeg M.A. (2015). Short-term administration of the glucagon receptor antagonist LY2409021 lowers blood glucose in healthy people and in those with type 2 diabetes. Diabetes Obes. Metab..

[7] Vajda E.G., Logan D., Lasseter K., Armas D., Plotkin D.J., Pipkin J.D., Li Y.X., Zhou R., Klein D., Wei X., Dilzer S., Zhi L., Marschke K.B. (2016). Pharmacokinetics and pharmacodynamics of single and multiple doses of the glucagon receptor antagonist LGD-6972 in healthy subjects and subjects with type 2 diabetes mellitus. Diabetes Obes. Metab..

[8] Briant L., Salehi A., Vergari E., Zhang Q., Rorsman P. (2016). Glucagon secretion from pancreatic alpha-cells. Ups J. Med. Sci..

[9] Hauge-Evans A.C., King A.J., Carmignac D., Richardson C.C., Robinson I.C., Low M.J., Christie M.R., Persaud S.J., Jones P.M. (2009). Somatostatin secreted by islet delta-cells fulfills multiple roles as a paracrine regulator of islet function. Diabetes.

[10] Strowski M.Z., Parmar R.M., Blake A.D., Schaeffer J.M. (2000). Somatostatin inhibits insulin and glucagon secretion via two receptors subtypes: An *in vitro* study of pancreatic islets from somatostatin receptor 2 knockout mice. Endocrinology.

[11] Franklin I.K., Wollheim C.B. (2004). GABA in the endocrine pancreas: Its putative role as an islet cell paracrine-signalling molecule. J. Gen. Physiol..

[12] Wendt A., Birnir B., Buschard K., Gromada J., Salehi A., Sewing S., Rorsman P., Braun M. (2004). Glucose inhibition of glucagon secretion from rat alpha-cells is mediated by GABA released from neighboring beta-cells. Diabetes.

[13] Franklin I., Gromada J., Gjinovci A., Theander S., Wollheim C.B. (2005). Beta-cell secretory products activate alpha-cell ATP-dependent potassium channels to inhibit glucagon release. Diabetes.

[14] Ishihara H., Maechler P., Gjinovci A., Herrera P.L., Wollheim C.B. (2003). Islet beta-cell secretion determines glucagon release from neighbouring alpha-cells. Nat. Cell Biol..

[15] Honey R.N., Weir G.C. (1980). Acetylcholine stimulates insulin, glucagon, and somatostatin release in the perfused chicken pancreas. Endocrinology.

[16] Duttaroy A., Zimliki C.L., Gautam D., Cui Y., Mears D., Wess J. (2004). Muscarinic stimulation of pancreatic insulin and glucagon release is abolished in m3 muscarinic acetylcholine receptor-deficient mice. Diabetes.

[17] Greenbaum C.J., Havel P.J., Taborsky G.J., Klaff L.J. (1991). Intra-islet insulin permits glucose to directly suppress pancreatic A cell function. J. Clin. Invest..

[18] Weir G.C., Knowlton S.D., Atkins R.F., McKennan K.X., Martin D.B. (1976). Glucagon secretion from the perfused pancreas of streptozotocin-treated rats. Diabetes.

[19] Maruyama H., Hisatomi A., Orci L., Grodsky G.M., Unger R.H. (1984). Insulin within islets is a physiologic glucagon release inhibitor. J. Clin. Invest..

[20] Kawamori D., Kurpad A.J., Hu J., Liew C.W., Shih J.L., Ford E.L., Herrera P.L., Polonsky K.S., McGuinness O.P., Kulkarni R.N. (2009). Insulin signaling in alpha cells modulates glucagon secretion *in vivo*. Cell Metab..

[21] McKinnon C.M., Ravier M.A., Rutter G.A. (2006). FoxO1 is required for the regulation of preproglucagon gene expression by insulin in pancreatic alphaTC1-9 cells. J. Biol. Chem..

[22] Akiyama M., Liew C.W., Lu S., Hu J., Martinez R., Hambro B., Kennedy R.T., Kulkarni R.N. (2013). X-box binding protein 1 is essential for insulin regulation of pancreatic alpha-cell function. Diabetes.

[23] Park S.W., Zhou Y., Lee J., Lu A., Sun C., Chung J., Ueki K., Ozcan U. (2010). The regulatory subunits of PI3K, p85alpha and p85beta, interact with XBP-1 and increase its nuclear translocation. Nat. Med..

[24] Winnay J.N., Boucher J., Mori M.A., Ueki K., Kahn C.R. (2010). A regulatory subunit of phosphoinositide 3-kinase increases the nuclear accumulation of X-box-binding protein-1 to modulate the unfolded protein response. Nat. Med..

[25] Taniguchi C.M., Emanuelli B., Kahn C.R. (2006). Critical nodes in signalling pathways: Insights into insulin action. Nat. Rev. Mol. Cell Biol..

[26] Withers D.J., Gutierrez J.S., Towery H., Burks D.J., Ren J.M., Previs S., Zhang Y., Bernal D., Pons S., Shulman G.I., Bonner-Weir S., White M.F. (1998). Disruption of IRS-2 causes type 2 diabetes in mice. Nature.

[27] Kulkarni R.N., Winnay J.N., Daniels M., Bruning J.C., Flier S.N., Hanahan D., Kahn C.R. (1999). Altered function of insulin receptor substrate-1-deficient mouse islets and cultured beta-cell lines. J. Clin. Invest..

[28] Takatani T., Shirakawa J., Roe M.W., Leech C.A., Maier B.F., Mirmira R.G., Kulkarni R.N. (2016). IRS1 deficiency protects beta-cells against ER stress-induced apoptosis by modulating sXBP-1 stability and protein translation. Sci. Rep..

[29] Kulkarni R.N., Roper M.G., Dahlgren G., Shih D.Q., Kauri L.M., Peters J.L., Stoffel M., Kennedy R.T. (2004). Islet secretory defect in insulin receptor substrate 1 null mice is linked with reduced calcium signaling and expression of sarco(endo)plasmic reticulum Ca2+-ATPase (SERCA)-2b and -3. Diabetes.

[30] Kubota N., Tobe K., Terauchi Y., Eto K., Yamauchi T., Suzuki R., Tsubamoto Y., Komeda K., Nakano R., Miki H., Satoh S., Sekihara H., Sciacchitano S., Lesniak M., Aizawa S. (2000). Disruption of insulin receptor substrate 2 causes type 2 diabetes because of liver insulin resistance and lack of compensatory beta-cell hyperplasia. Diabetes.

[31] Kubota N., Kubota T., Kajiwara E., Iwamura T., Kumagai H., Watanabe T., Inoue M., Takamoto I., Sasako T., Kumagai K., Kohjima M., Nakamuta M., Moroi M., Sugi K., Noda T. (2016). Differential hepatic distribution of insulin receptor substrates causes selective insulin resistance in diabetes and obesity. Nat. Commun..

[32] Le Marchand S.J., Piston D.W. (2010). Glucose suppression of glucagon secretion: Metabolic and calcium responses from alpha-cells in intact mouse pancreatic islets. J. Biol. Chem..

[33] Park K., Li Q., Rask-Madsen C., Mima A., Mizutani K., Winnay J., Maeda Y., D'Aquino K., White M.F., Feener E.P., King G.L. (2013). Serine phosphorylation sites on IRS2 activated by angiotensin II and protein kinase C to induce selective insulin resistance in endothelial cells. Mol. Cell Biol..

[34] Bozadjieva N., Blandino-Rosano M., Chase J., Dai X.Q., Cummings K., Gimeno J., Dean D., Powers A.C., Gittes G.K., Ruegg M.A., Hall M.N., MacDonald P.E., Bernal-Mizrachi E. (2017). Loss of mTORC1 signaling alters pancreatic alpha cell mass and impairs glucagon secretion. J. Clin. Invest..

[35] Liew C.W., Assmann A., Templin A.T., Raum J.C., Lipson K.L., Rajan S., Qiang G., Hu J., Kawamori D., Lindberg I., Philipson L.H., Sonenberg N., Goldfine A.B., Stoffers D.A., Mirmira R.G. (2014). Insulin regulates carboxypeptidase E by modulating translation initiation scaffolding protein eIF4G1 in pancreatic beta cells. Proc. Natl. Acad. Sci. U. S. A..

[36] Rorsman P., Braun M., Zhang Q. (2012). Regulation of calcium in pancreatic alpha- and beta-cells in health and disease. Cell Calcium.

[37] Gazit N., Vertkin I., Shapira I., Helm M., Slomowitz E., Sheiba M., Mor Y., Rizzoli S., Slutsky I. (2016). IGF-1 receptor differentially regulates spontaneous and evoked transmission via mitochondria at hippocampal synapses. Neuron.

[38] Liu S.M., Okada T., Assmann A., Soto J., Liew C.W., Bugger H., Shirihai O.S., Abel E.D., Kulkarni R.N. (2009). Insulin signaling regulates mitochondrial function in pancreatic beta-cells. PLoS One.

[39] Cryer P.E. (2012). Minireview: Glucagon in the pathogenesis of hypoglycemia and hyperglycemia in diabetes. Endocrinology.

[40] Araujo E.P., Amaral M.E., Souza C.T., Bordin S., Ferreira F., Saad M.J., Boschero A.C., Magalhaes E.C., Velloso L.A. (2002). Blockade of IRS1 in isolated rat pancreatic islets improves glucose-induced insulin secretion. FEBS Lett..

[41] Vergari E., Knudsen J.G., Ramracheya R., Salehi A., Zhang Q., Adam J., Asterholm I.W., Benrick A., Briant L.J.B., Chibalina M.V., Gribble F.M., Hamilton A., Hastoy B., Reimann F., Rorsman N.J.G. (2019). Insulin inhibits glucagon release by SGLT2-induced stimulation of somatostatin secretion. Nat. Commun..

[42] Zhou A., Pacini G., Ahren B., D'Argenio D.Z. (2014). Glucagon clearance is regulated by nutritional state: Evidence from experimental studies in mice. Diabetologia.

[43] Okada T., Liew C.W., Hu J., Hinault C., Michael M.D., Krtzfeldt J., Yin C., Holzenberger M., Stoffel M., Kulkarni R.N. (2007). Insulin receptors in beta-cells are critical for islet compensatory growth response to insulin resistance. Proc. Natl. Acad. Sci. U. S. A..

[44] Kulkarni R.N., Bruning J.C., Winnay J.N., Postic C., Magnuson M.A., Kahn C.R. (1999). Tissue-specific knockout of the insulin receptor in pancreatic beta cells creates an insulin secretory defect similar to that in type 2 diabetes. Cell.

[45] Shen X.X., Li H.L., Pan L., Hong J., Xiao J., Hermansen K., Jeppesen P.B., Li G.W. (2012). Glucotoxicity and alpha cell dysfunction: Involvement of the PI3K/Akt pathway in glucose-induced insulin resistance in rat islets and clonal alphaTC1-6 cells. Endocr. Res..

[46] da Silva Xavier G., Farhan H., Kim H., Caxaria S., Johnson P., Hughes S., Bugliani M., Marselli L., Marchetti P., Birzele F., Sun G., Scharfmann R., Rutter J., Siniakowicz K., Weir G. (2011). Per-arnt-sim (PAS) domain-containing protein kinase is downregulated in human islets in type 2 diabetes and regulates glucagon secretion. Diabetologia.

[47] Tang C.Y., Man X.F., Guo Y., Tang H.N., Tang J., Zhou C.L., Tan S.W., Wang M., Zhou H.D. (2017). IRS-2 partially compensates for the insulin signal defects in IRS-1(-/-) mice mediated by miR-33. Mol. Cells.

[48] Ozoe A., Sone M., Fukushima T., Kataoka N., Arai T., Chida K., Asano T., Hakuno F., Takahashi S. (2013). Insulin receptor substrate-1 (IRS-1) forms a ribonucleoprotein complex associated with polysomes. FEBS Lett..

[49] Zhang Q., Ramracheya R., Lahmann C., Tarasov A., Bengtsson M., Braha O., Braun M., Brereton M., Collins S., Galvanovskis J., Gonzalez A., Groschner L.N., Rorsman N.J., Salehi A., Travers M.E. (2013). Role of KATP channels in glucose-regulated glucagon secretion and impaired counterregulation in type 2 diabetes. Cell Metab..

[50] Aspinwall C.A., Qian W.J., Roper M.G., Kulkarni R.N., Kahn C.R., Kennedy R.T. (2000). Roles of insulin receptor substrate-1, phosphatidylinositol 3-kinase, and release of intracellular Ca2+ stores in insulin-stimulated insulin secretion in beta -cells. J. Biol. Chem..

[51] Kim M.S., Hong J.H., Li Q., Shin D.M., Abramowitz J., Birnbaumer L., Muallem S. (2009). Deletion of TRPC3 in mice reduces store-operated Ca2+ influx and the severity of acute pancreatitis. Gastroenterology.

[52] Abels M., Riva M., Bennet H., Ahlqvist E., Dyachok O., Nagaraj V., Shcherbina L., Fred R.G., Poon W., Sorhede-Winzell M., Fadista J., Lindqvist A., Kask L., Sathanoori R., Dekker-Nitert M. (2016). CART is overexpressed in human type 2 diabetic islets and inhibits glucagon secretion and increases insulin secretion. Diabetologia.

[53] Edlund A., Esguerra J.L., Wendt A., Flodstrom-Tullberg M., Eliasson L. (2014). CFTR and anoctamin 1 (ANO1) contribute to cAMP amplified exocytosis and insulin secretion in human and murine pancreatic beta-cells. BMC Med..

[54] Allister E.M., Robson-Doucette C.A., Prentice K.J., Hardy A.B., Sultan S., Gaisano H.Y., Kong D., Gilon P., Herrera P.L., Lowell B.B., Wheeler M.B. (2013). UCP2 regulates the glucagon response to fasting and starvation. Diabetes.

[55] Riehle C., Wende A.R., Zhu Y., Oliveira K.J., Pereira R.O., Jaishy B.P., Bevins J., Valdez S., Noh J., Kim B.J., Moreira A.B., Weatherford E.T., Manivel R., Rawlings T.A., Rech M. (2014). Insulin receptor substrates are essential for the bioenergetic and hypertrophic response of the heart to exercise training. Mol. Cell Biol..

[56] Kusminski C.M., Chen S., Ye R., Sun K., Wang Q.A., Spurgin S.B., Sanders P.E., Brozinick J.T., Geldenhuys W.J., Li W.H., Unger R.H., Scherer P.E. (2016). MitoNEET-parkin effects in pancreatic alpha- and beta-cells, cellular survival, and intrainsular cross talk. Diabetes.

[57] Dong X.C., Copps K.D., Guo S., Li Y., Kollipara R., DePinho R.A., White M.F. (2008). Inactivation of hepatic Foxo1 by insulin signaling is required for adaptive nutrient homeostasis and endocrine growth regulation. Cell Metab..

[58] Lin X., Taguchi A., Park S., Kushner J.A., Li F., Li Y., White M.F. (2004). Dysregulation of insulin receptor substrate 2 in beta cells and brain causes obesity and diabetes. J. Clin. Invest..

[59] Herrera P.L. (2000). Adult insulin- and glucagon-producing cells differentiate from two independent cell lineages. Development.

[60] Shiota C., Prasadan K., Guo P., Fusco J., Xiao X., Gittes G.K. (2017). Gcg (CreERT2) knockin mice as a tool for genetic manipulation in pancreatic alpha cells. Diabetologia.

[61] Nishiki Y., Adewola A., Hatanaka M., Templin A.T., Maier B., Mirmira R.G. (2013). Translational control of inducible nitric oxide synthase by p38 MAPK in islet beta-cells. Mol. Endocrinol..

