# Supplementary material for: Insulin receptor substrate 1, but not IRS2, plays a dominant role in regulating pancreatic alpha cell function in mice
Source: J Biol Chem. 2021 Apr 9;296:100646. doi: 10.1016/j.jbc.2021.100646 (PMC8131928; doi:10.1016/j.jbc.2021.100646)
Supplement: Supplemental Figures S1–S11 [file mmc1.pdf]

Fig. S1

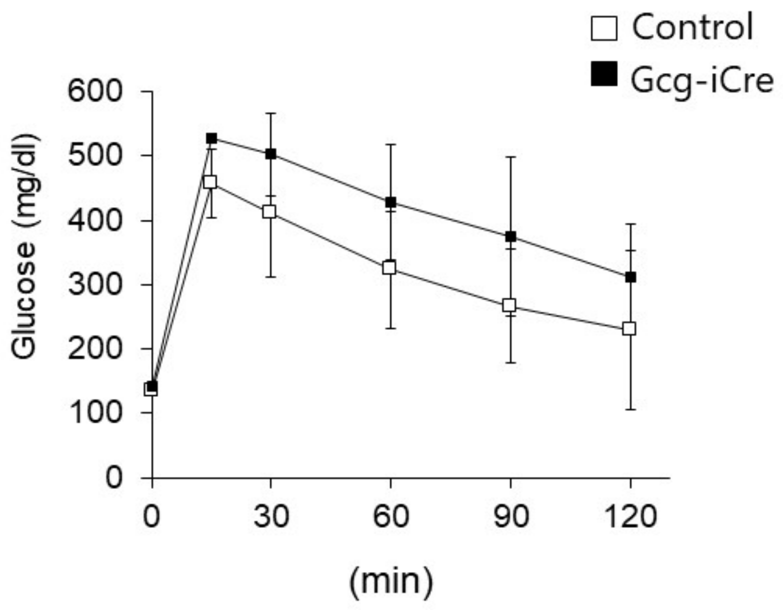

Fig. S1 Glucagon-cre transgene does not significantly affect glucose tolerance.

Oral glucose tolerance test (OGTT; glucose 1 g/kg body weight) in Control (wild type ;n= 3) and Gcg-iCre mice (n=3).

Fig. S2

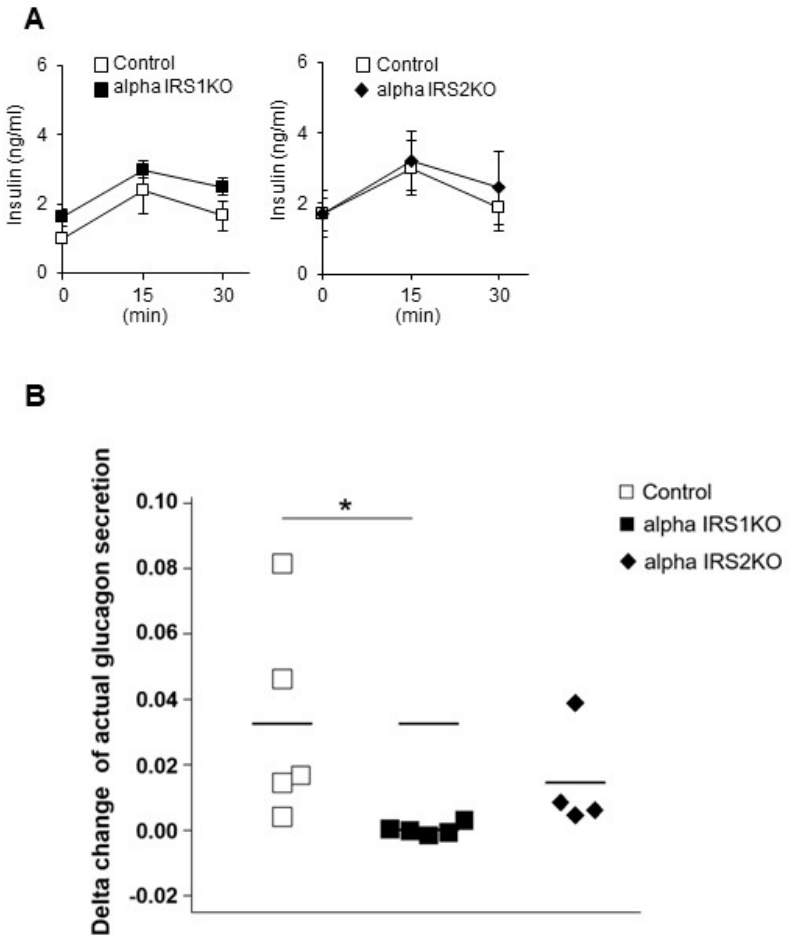

A
Control
alpha IRS1KO
Insulin (ng/ml)
0
2
4
6
0
15
30
(min)
Control
alpha IRS2KO
Insulin (ng/ml)
0
2
4
6
0
15
30
(min)
B
Delta change of actual glucagon secretion
0.10
0.08
0.06
0.04
0.02
0.00
-0.02
*
Control
alpha IRS1KO
alpha IRS2KO

Fig. S2.

Alpha cell specific deletion of IRS1 or IRS2 does not significantly affect insulin levels in OGTT.

(A) Serum Insulin levels of OGTT measured by ELISA in 6-month-old alpha IRS1KO (n = 5 in each group) or alphaIRS2KO (n = 3-4 in each group) mice. (B) Plot of individual data points from Fig. 1G. The panel shows the data pointes of actual glucose value. Data are expressed as means ± SD * $p<0.05$.

Fig.S3

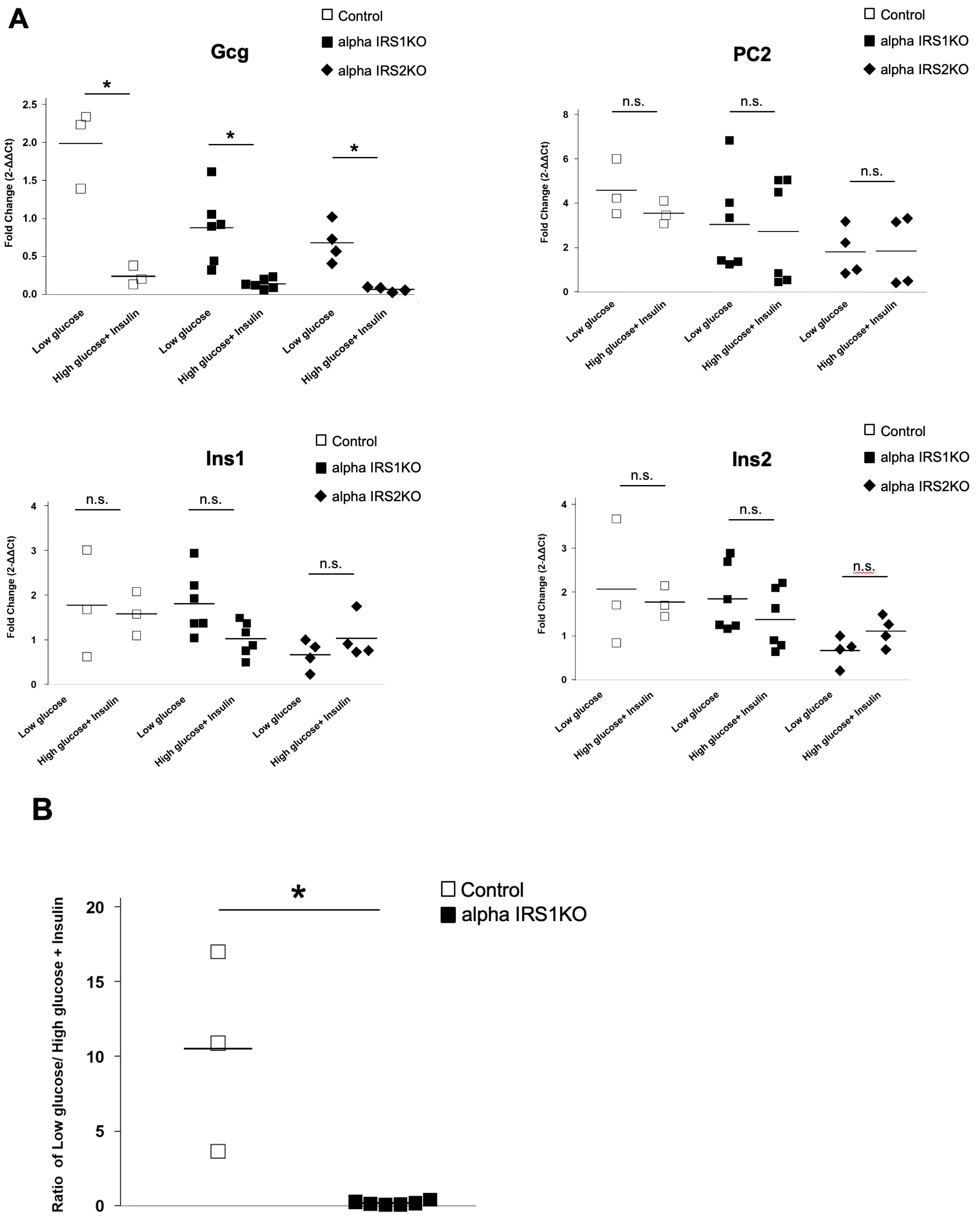
A
Gcg
PC2
Ins1
Ins2
Control
alpha IRS1KO
alpha IRS2KO
Fold Change (2-ΔΔCt)
Low glucose
High glucose+ Insulin
*
n.s.
B
Ratio of Low glucose/ High glucose + Insulin
Control
alpha IRS1KO
*

Fig. S3

Islets with alpha cell specific deletion of IRS1 do not significantly suppress glucagon transcription in response to insulin.

(A) qRT-PCR quantification of glucagon, ins1, ins2 and PC2 expression normalized by beta-actin. Forty islets from control mice, alphaIRS1KO mice and alphaIRS2KO mice were used for each experiment. Data are expressed as means ± SD. Gcg:Two-way ANOVA, F= 7.404, $p=1.218\times10^{-2}$ (IRS), F= 20.868, $p=1.370\times10^{-4}$ (Insulin), F= 1.898, p=0.182 (Interaction), PC2: Two-way ANOVA, F= 1.283, p=0.269 (IRS), F=3.496, p=0.074 (Insulin), F= 0.223, p=0.641 (Interaction), Ins1:Two-way ANOVA, F= 1.748, p=0.199 (IRS), F= 8.778, $p=6.970\times10^{-3}$ (Insulin), F= 0.096, p=0.759 (Interaction), Ins2:Two-way ANOVA, F= 3.159, p=0.089 (IRS), F= 10.030, p=0.004 (Insulin), F= 0.626, p=0.437 (Interaction),*p<0.05 (B) Ratio of glucagon expression of low glucose (3.3 mM) to high glucose (16.7 mM)+insulin (100 nM) condition. Data are expressed as means ± SD. *p<0.05

Fig.S4

A

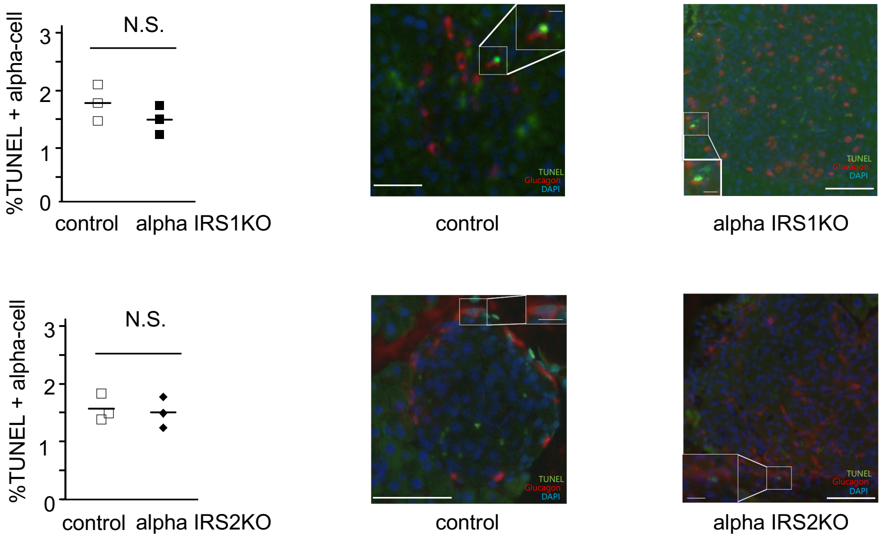
%TUNEL + alpha-cell
N.S.
0
1
2
3
control
alpha IRS1KO
TUNEL
Glucagon
DAPI
control
alpha IRS1KO
%TUNEL + alpha-cell
N.S.
0
1
2
3
control
alpha IRS2KO
control
alpha IRS2KO

B

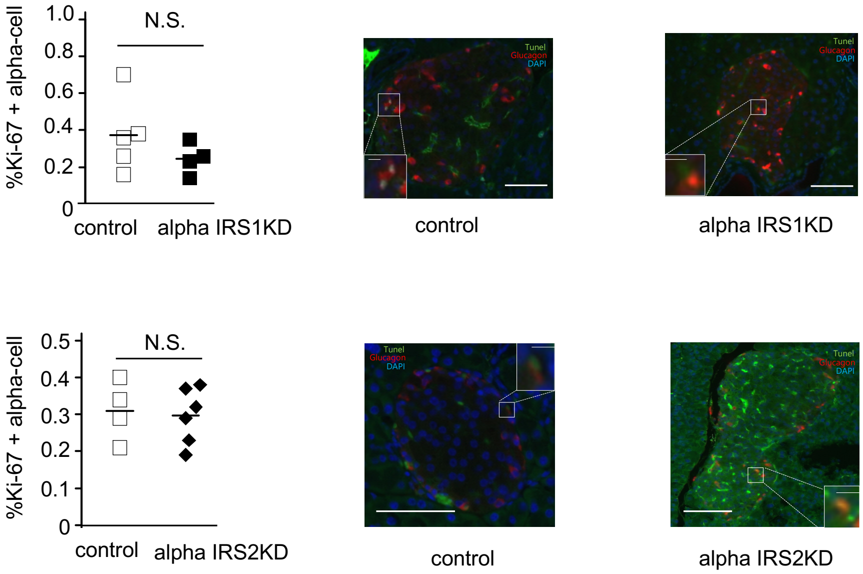
%Ki-67 + alpha-cell
N.S.
0
0.2
0.4
0.6
0.8
1.0
control
alpha IRS1KD
Tunel
Glucagon
DAPI
control
alpha IRS1KD
%Ki-67 + alpha-cell
N.S.
0
0.1
0.2
0.3
0.4
0.5
control
alpha IRS2KD
control
alpha IRS2KD

Fig. S4.

(A,B) Alpha cell specific deletion of IRS1 or IRS2 does not significantly affect alpha cell apoptosis or proliferation. Percent TUNEL Ki-67 positive alpha cells in pancreas sections from alpha IRS1KO or alpha IRS2KO mice (n=3). (B) Percent Ki67 positive alpha cells in pancreas sections from alpha IRS1KO or alpha IRS2KO mice (n=4-6 in each group). Between 500-1000 cells from multiple sections of pancreas were counted in all groups. Data are expressed as means ± SD. Representative images are shown in right panel. Scale bars are 50 μm in low magnification and 10 μm in high magnification insets.

Fig.S5

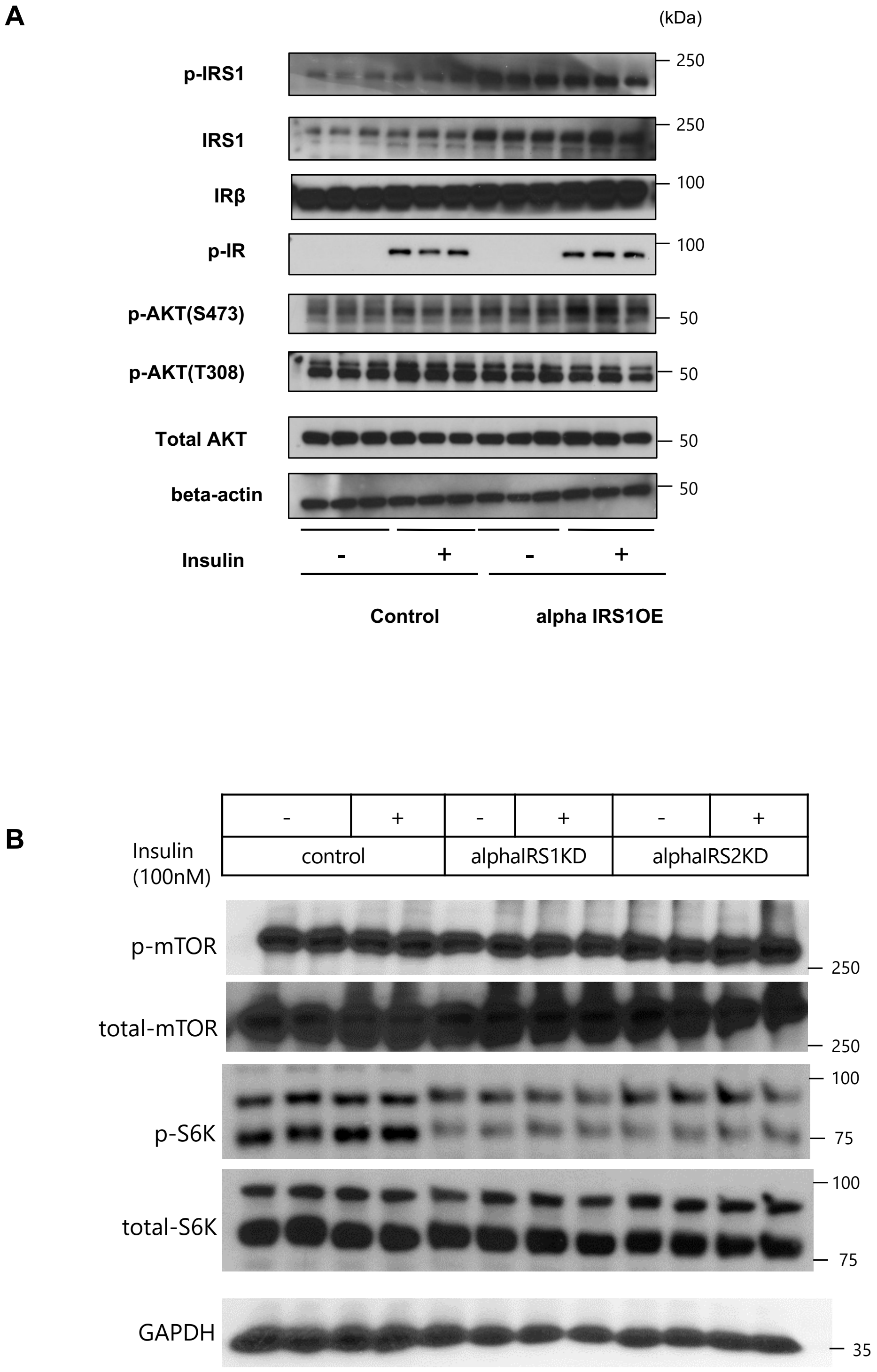

A
(kDa)
p-IRS1
250
IRS1
250
IRβ
100
p-IR
100
p-AKT(S473)
50
p-AKT(T308)
50
Total AKT
50
beta-actin
50
Insulin
-
+
-
+
Control
alpha IRS1OE
B
Insulin
(100nM)
-
+
-
+
-
+
control
alphaIRS1KD
alphaIRS2KD
p-mTOR
250
total-mTOR
250
p-S6K
100
75
total-S6K
100
75
GAPDH
35

C

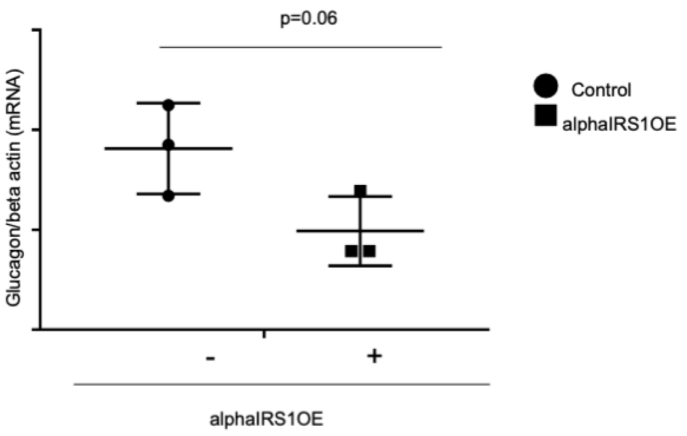

Fig. S5

IRS1 over-expression in alpha cells affects glucagon mRNA expression.

(A) Western blotting for IRS1, IRS1 Ser^{307} phosphorylation, insulin receptor (IR) tyrosine ^{972} phosphorylation (pIR), AKT Thr^{308} phosphorylation, AKT Ser^{473} phosphorylation, total AKT protein, and beta-actin in control or alpha IRS1OE cells. (B) Western blotting for p-S6K (Ser371) and total S6K in control, alpha IRS1KD and alpha IRS2 KD cells. (C) PCR for glucagon gene expression in control or alphaIRS1OE cells (n=3). Data are expressed as means ± SD.

Fig. S6

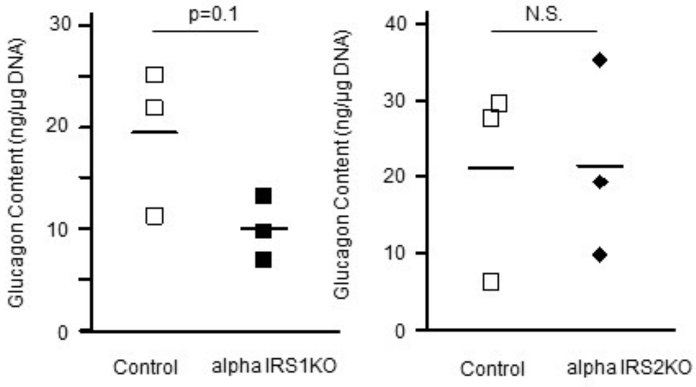

Fig. S6.

Glucagon content shows a trend to decrease in islets isolated from alpha IRS1KO mice.

Glucagon content in islets isolated from alphaIRS1KO (n=3) or alphaIRS2KO mice (n=3). Data are expressed as means ± SD.

Fig. S7

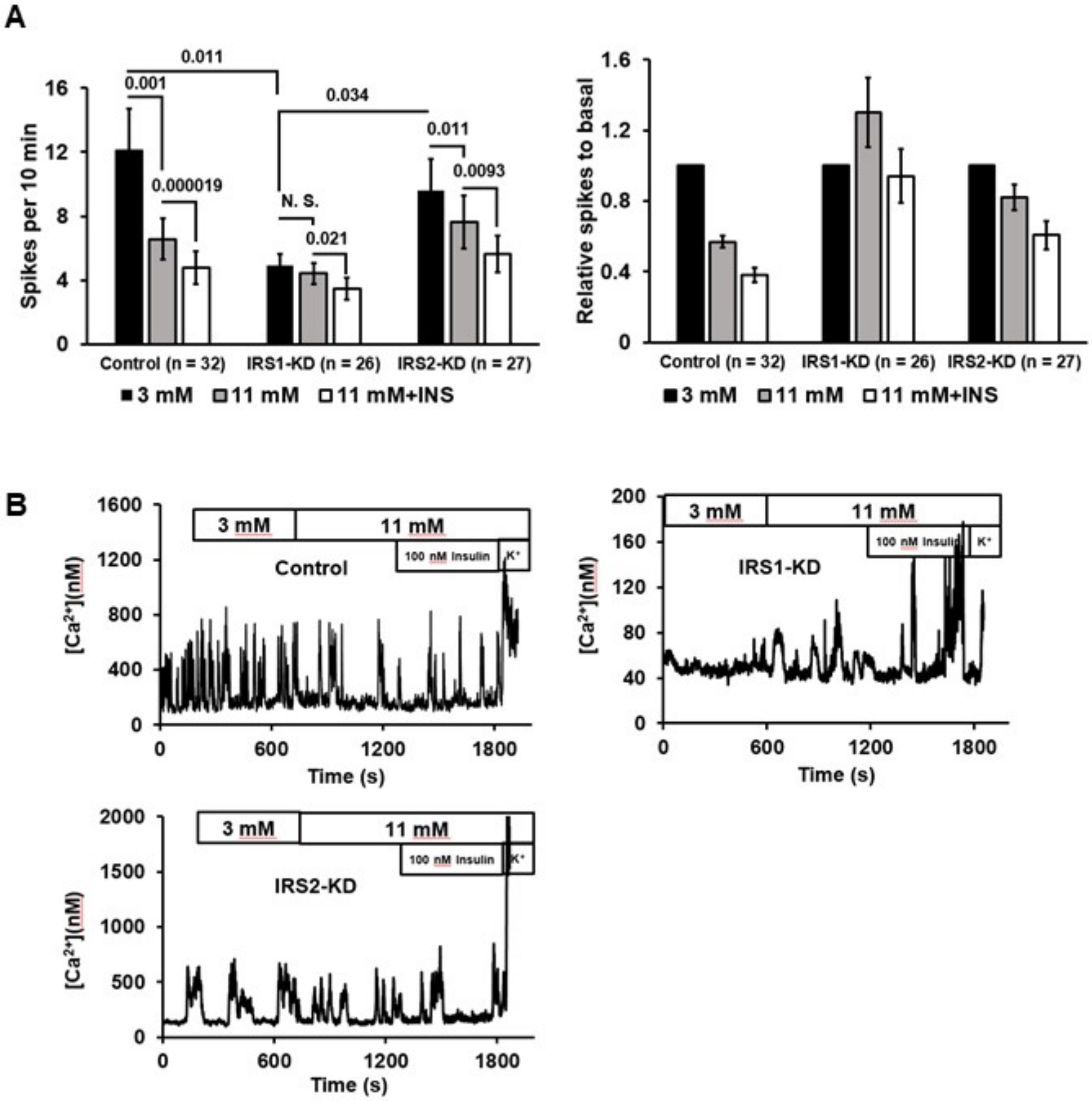

Fig. S7

Fig. S7. AlphaIRS1KD cells exhibit insufficient suppression of Ca^{2+} -influx in response to high glucose

(A) Control, alphaIRS1KD or alphaIRS2KD cells were perfused with 3 mM glucose, 11 mM glucose and 11 mM glucose + 100 nM insulin for 10 min during each period. The cells were imaged to measure [Ca^{2+}] influx. The data normalized by dividing all the spike numbers to their basal level

are also shown. (B) The representative [Ca^{2+}] traces of control, alphaIRS1KD or alphaIRS2KD cells.

Fig.S8

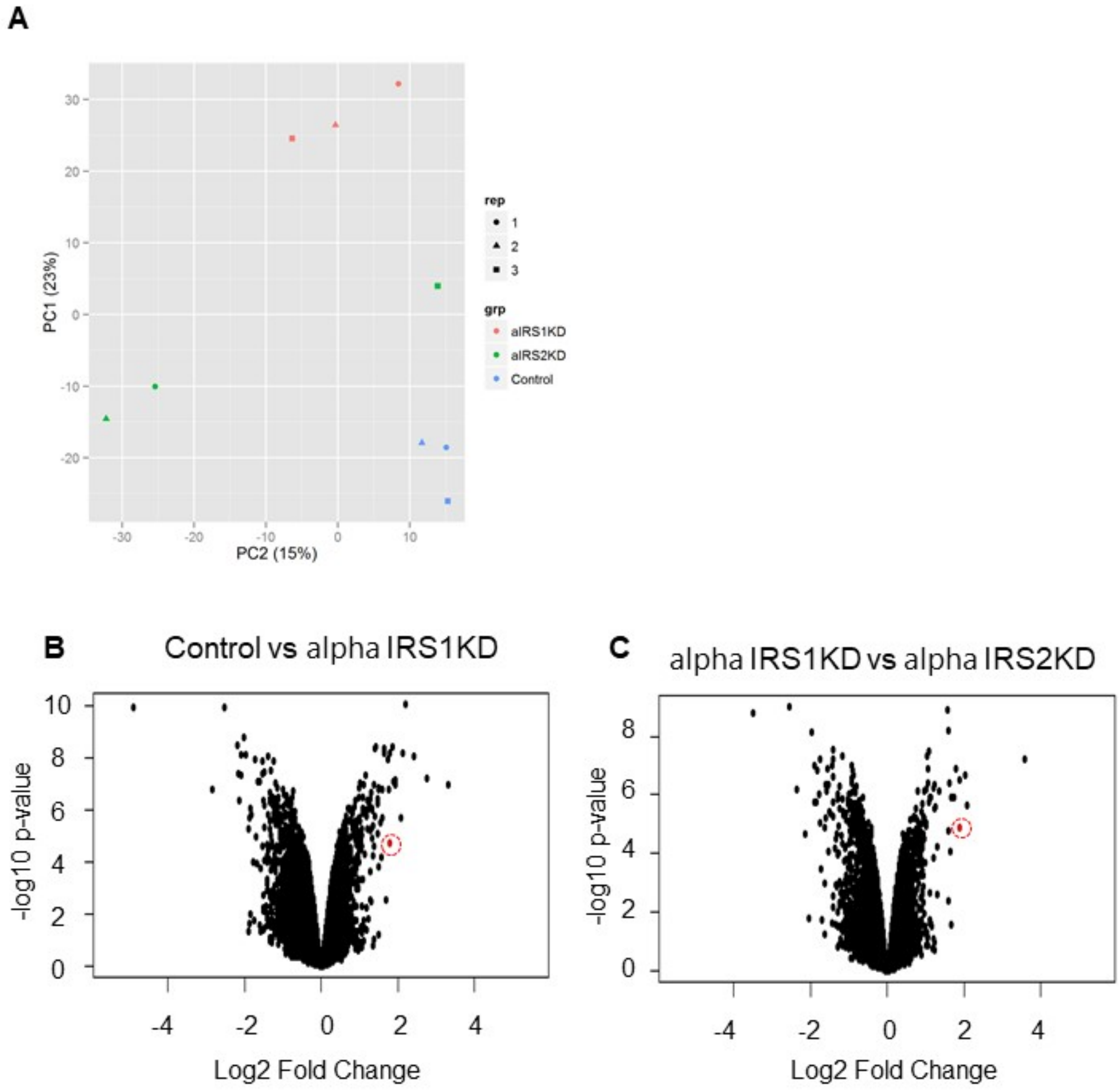
A
PC1 (23%)
PC2 (15%)
30
20
10
0
-10
-20
-30
-20
-10
0
10
rep
1
2
3
grp
aIRS1KD
aIRS2KD
Control
B
Control vs alpha IRS1KD
-log10 p-value
Log2 Fold Change
10
8
6
4
2
0
-4
-2
0
2
4
C
alpha IRS1KD vs alpha IRS2KD
-log10 p-value
Log2 Fold Change
8
6
4
2
0
-4
-2
0
2
4

Fig. S8.

Microarray analysis of control, alphaIRS1KD or alphaIRS2KD alpha cells.

(A) Principal component (PC) analysis for control, alphaIRS1KD, and alphaIRS2KD cells. (B and C) A volcano plot showing comparison of gene expression for control and alpha IRS1KD (n=3) (B) or alpha IRS1KD and alpha IRS2KD samples (C) (n=3). The red dot in each plot represents the protein Ano1 (circled in red).

Fig. S9

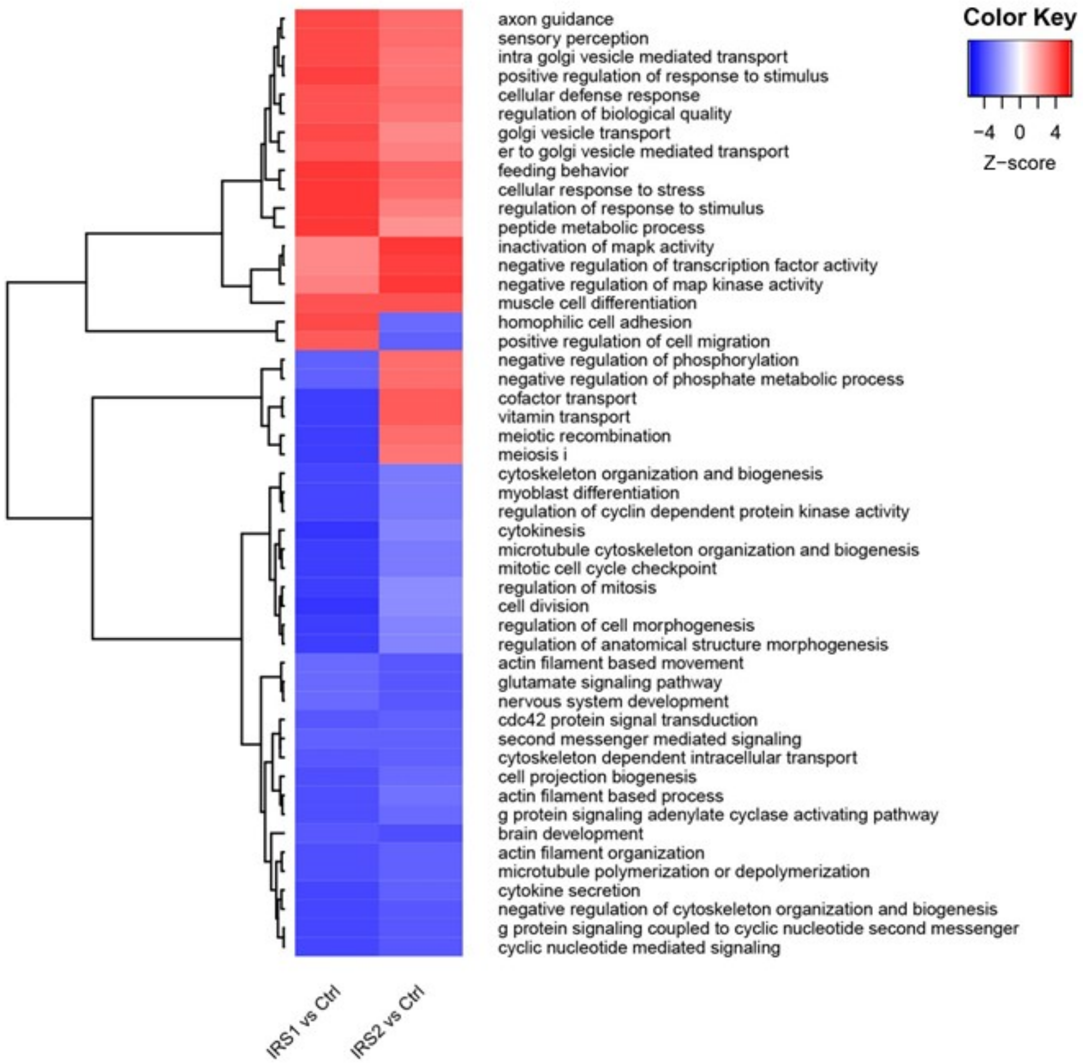
Color Key
-4 0 4
Z-score
axon guidance
sensory perception
intra golgi vesicle mediated transport
positive regulation of response to stimulus
cellular defense response
regulation of biological quality
golgi vesicle transport
er to golgi vesicle mediated transport
feeding behavior
cellular response to stress
regulation of response to stimulus
peptide metabolic process
inactivation of mapk activity
negative regulation of transcription factor activity
negative regulation of map kinase activity
muscle cell differentiation
homophilic cell adhesion
positive regulation of cell migration
negative regulation of phosphorylation
negative regulation of phosphate metabolic process
cofactor transport
vitamin transport
meiotic recombination
meiosis i
cytoskeleton organization and biogenesis
myoblast differentiation
regulation of cyclin dependent protein kinase activity
cytokinesis
microtubule cytoskeleton organization and biogenesis
mitotic cell cycle checkpoint
regulation of mitosis
cell division
regulation of cell morphogenesis
regulation of anatomical structure morphogenesis
actin filament based movement
glutamate signaling pathway
nervous system development
cdc42 protein signal transduction
second messenger mediated signaling
cytoskeleton dependent intracellular transport
cell projection biogenesis
actin filament based process
g protein signaling adenylate cyclase activating pathway
brain development
actin filament organization
microtubule polymerization or depolymerization
cytokine secretion
negative regulation of cytoskeleton organization and biogenesis
g protein signaling coupled to cyclic nucleotide second messenger
cyclic nucleotide mediated signaling
IRS1 vs Ctrl
IRS2 vs Ctrl

Fig. S9

Heatmap of microarray analysis of control, alpha IRS1KD or alpha IRS2KD alpha cells.

A heatmap showing a comparison of gene expression for control and alphaIRS1KD (n=3) or control and alphaIRS2KD (n=3). The “cellular response to stress” pathway was increased in alpha IRS1KD compared to control and alphaIRS2KD and “negative regulation of phosphorylation and phosphate metabolic process” pathways were increased in alphaIRS1KD alpha cells.

Fig. S10

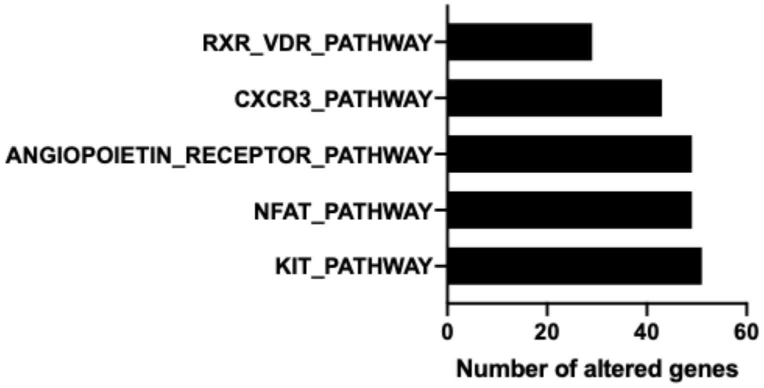

RXR_VDR pathway		
**Gene-Entrez #**	**Gene abbreviation**	**Gene name**
11303	Abca1	ATP-binding cassette, sub-family A (ABC1), member 1
15370	Nr4a1	nuclear receptor subfamily 4, group A, member 1

CXCR3 pathway		
**Gene-Entrez #**	**Gene abbreviation**	**Gene name**
19094	Mapk11	mitogen-activated protein kinase 11

Angiopoietin receptor pathway		
**Gene-Entrez #**	**Gene abbreviation**	**Gene name**
23871	Ets1	E26 avian leukemia oncogene 1, 5' domain
20850	Stat5a	signal transducer and activator of transcription 5A

NFAT pathway		
**Gene-Entrez #**	**Gene abbreviation**	**Gene name**
19087	Prkar2a	protein kinase, cAMP dependent regulatory, type II alpha
11459	Acta1	actin, alpha 1, skeletal muscle

KIT pathway		
**Gene-Entrez #**	**Gene abbreviation**	**Gene name**
16590	Kit	KIT proto-oncogene receptor tyrosine kinase
17444	Grap2	GRB2-related adaptor protein 2

Fig. S10. Pathway analysis of alphaIRS1KD cells linked with mTOR-S6K signaling

Upper panel shows pathways linked with mTOR-S6K signaling that are significantly altered in alphaIRS1KD cells compared to control cells. Lower tables show the representative transcription factors in each pathway.

Fig.S11

Primer name	Sequence (5' to 3')
Tapbp_Fw	GCTGCCTACTGGACCATTCC
Tapbp_Rv	AGGGGCTACTGGAGTCATCT
Pam_Fw	GGAGCCTCCAACTAACTCCG
Pam_Rv	GAGCAGGGTCCTTATCGCTC
Tpp1_Fw	AGCATGGGACAGGACTCTTTG
Tpp1_Rv	CTGCTTTCTCGTCATGGCAC
Scg5_Fw	GAGGTTTCGGAGGGAGGTGA
Scg5_Rv	CCAGCCTTGAGGCCATTTTT
Cartpt_Fw	AGGAGCTGATCGAAGCGTTG
Cartpt_Rv	GGGACTTGGCCGTACTTCTT
Snap25_Fw	ATGTTGGATGAGCAAGGCGA
Snap25_Rv	TGGCCACTACTCCATCCTGA
Eif2ak3_Fw	GGGTCTGGTTCCTTGGTTTCA
Eif2ak3_Rv	TCATCCCAACACACGCTCA
Apbb1_Fw	CTGGGCCACCTTATCACAGG
Apbb1_Rv	CCAGCCGGTAGATCGGAATC
Trpc3_Fw	ACCCTTCAGTCTGGTCCCTA
Trpc3_Rv	TCGAGTTAGACTGTGTGAAGAGG
Cacna2d1_Fw	CGAACATACACATGGACGCC
Cacna2d1_Rv	GGGTTTCTGAATATCTGGCCT

Fig. S11.

qRT-PCR Primer sequences. Related to Fig. 6C
